# Examining Computer-Science Students’ Metacognitive Beliefs and Reported Practices in Verifying AI-Generated Code: A Concurrent Mixed-Methods Study

**DOI:** 10.3390/jintelligence14100232

**Published:** 2026-10-01

**Authors:** Shanshan Li, Jingqi Tang, Zhen Qiang, Zhuo Wang

**Affiliations:** 1 School of Marxism, Chengdu Sport University, Chengdu 641418, China; 2 College of Educational Sciences, Qingdao University, Qingdao 266071, China; 3 School of Foreign Languages, Lanzhou Jiaotong University, Lanzhou 730070, China

**Keywords:** generative AI, AI-generated code, verification, computer science, undergraduate, cross-sectional survey

## Abstract

Generative artificial intelligence (GenAI) accelerates programming, but its educational value depends on whether students check generated code before accepting it. This concurrent mixed-methods study examined which beliefs accompany such checking among computer-science students at one Chinese university. Survey responses from 294 students were analyzed with an exploratory structural equation model treating three practices as separate outcomes: checking code line by line before use, practicing without AI, and declining in advance to delegate. Robustness checks included ordinal estimation, acquiescence modeling, split-half replication, and multiverse analysis; eleven students were interviewed. Verification-dispensability belief, endorsing reasons for treating further checking as unnecessary, was the only construct negatively associated with all three practices in every robustness check. Other beliefs were narrower or inert: perceived AI literacy accompanied line-by-line checking alone, generic metacognitive self-regulation none. Delegation breadth, the number of activities handed to GenAI, was unrelated to all three: volume of delegation said little about checking. Interviews exposed a mismatch between self-reported categories and actual workflows: students allocated checking by task purpose, accepted process-level understanding, and delegated verification to other AI models, a practice unmeasured by the survey. The data are cross-sectional and self-reported, so these are associations requiring behavioral and longitudinal confirmation.

## 1. Introduction

Programming has become one of the most consequential domains of generative artificial intelligence (GenAI). Code-oriented large language models can translate natural-language intentions into executable programs, complete functions, explain unfamiliar syntax, generate tests, and assist with debugging. These capabilities change both the speed and the distribution of work in software development. Observational research describes programmers moving between an acceleration mode, in which an AI assistant implements an already understood plan, and an exploration mode, in which the assistant helps define what to do next (Barke et al., 2023). Controlled studies likewise show that code generators can lower barriers for novice learners and support task completion (Kazemitabaar et al., 2023). For computing education, this is not a marginal tool change. It alters which parts of programming students perform themselves, which parts they delegate, and which forms of knowledge remain visible to teachers and learners (Becker et al., 2023; Denny et al., 2024).

The same properties that make GenAI useful also make its output difficult to govern. Generated code may compile, pass visible tests, and appear idiomatic while remaining insecure, brittle, non-reproducible, or wrong under untested conditions. Empirical evaluations have found security weaknesses in code suggestions (Pearce et al., 2022), more insecure solutions from some users working with AI assistance (Perry et al., 2023), and apparently plausible but buggy or non-optimal solutions that novices may be ill-equipped to filter (Moradi Dakhel et al., 2023). Even when multiple suggestions are available, selecting among them entails non-trivial checking costs (Oertel et al., 2025). Consequently, the educational question is not simply whether students use GenAI, but whether they preserve epistemic control over code whose production has been partly delegated.

Verification is central to that control. In professional software engineering, code review is not reducible to executing a program: reviewers seek to understand changes, identify defects, consider alternatives, and share knowledge (Bacchelli & Bird, 2013; Sadowski et al., 2018). In programming education, comprehending and tracing code are themselves foundational competencies (Lister et al., 2004). GenAI complicates both practices because the system can produce a complete, fluent solution before the learner has formed a mental model of the problem. Execution success can then serve as a practical criterion for ending inspection: if the code runs or visible tests pass, further checking may appear unnecessary. Yet successful execution is evidence about a bounded set of inputs, not proof that the learner understands the code or that the code is correct, secure, maintainable, and transferable to a new context.

Computer-science undergraduates are a theoretically and practically important population in which to study this problem. They are still developing code comprehension, debugging, and design knowledge, but many are also approaching internships and programming-intensive employment. AI assistance therefore operates simultaneously as a learning scaffold and a prospective workplace tool. Existing studies have provided important evidence from introductory programmers (Kazemitabaar et al., 2023; Prather et al., 2024), professional or mixed-expertise developers (Barke et al., 2023; Mozannar et al., 2024), and programming instructors adapting their courses to AI code generation (Lau & Guo, 2023). Less is known about how degree-seeking computer-science students, especially outside Western settings, form beliefs about what counts as sufficient verification and convert those beliefs into task-specific decisions.

This distinction between beliefs and decisions matters because self-reported trust, literacy, or self-regulation may not map directly onto behavior. Trust in automation is useful only when it is calibrated to actual capability and task conditions (Lee & See, 2004). Automation research shows that people can omit checks or accept imperfect recommendations even when they have received training or possess relevant expertise (Parasuraman & Manzey, 2010). Research on AI-assisted decision making similarly distinguishes a general attitude toward AI from appropriate reliance on a case-by-case basis (Schemmer et al., 2023). In programming, a student may claim to be critical of AI while treating execution success as sufficient in practice, or may regulate learning efficiently by improving prompts and delegating more work rather than by inspecting code more deeply. A generic measure of self-regulated learning may therefore miss the domain-specific monitoring judgment that prompts or ends checking.

The present study addresses this problem with a convergent parallel, concurrent mixed-methods design. Because the survey measures were retrospective self-reports collected at one time point, the quantitative strand is exploratory and describes associations rather than causal processes. The following research questions were addressed:

**RQ1.**  *How broadly do students delegate programming-related tasks to GenAI, how often do they report three verification-related practices (line-by-line comprehension before use, independent practice without AI, and anticipatory non-delegation), and do these reports differ by gender and study level?*

**RQ2.**  *Which beliefs are associated with each of the three practices, and which associations remain stable across response-style control, alternative estimators and specifications, and random half-samples?*

**RQ3.**  *How do students describe what counts as sufficient checking of AI-generated code, and how do their accounts confirm, qualify, conflict with, or extend the survey associations?*

## 2. Literature Review

### 2.1. GenAI in Programming: Capability, Productivity, and Educational Opportunity

AI code generation spans code completion, function synthesis, explanation, debugging, refactoring, test generation, and documentation. These affordances can reduce the cost of translating an idea into executable form and make unfamiliar libraries or languages more accessible. In a grounded study of GitHub Copilot use, Barke et al. (2023) showed that programmers use generated suggestions both to accelerate known work and to explore uncertain solution spaces. Usability studies nevertheless found that the time saved in code production can be offset by difficulties understanding, editing, and validating generated suggestions (Vaithilingam et al., 2022). More recent behavioral modeling likewise treats verification as a cost-bearing component of AI-assisted programming, rather than assuming that suggestion acceptance is equivalent to productivity (Mozannar et al., 2024).

For novice learners, code generators can provide examples, explanations, and immediate feedback that would otherwise require instructor or peer support. Kazemitabaar et al. (2023) found that novices could benefit from AI code generation without uniformly abandoning learning-oriented engagement. However, the educational value of a generated solution depends on how it is used. A learner may compare it with an independent attempt, trace its logic, or use it to diagnose an error; alternatively, the learner may substitute the output for the cognitive work that the task was designed to elicit. Computing-education scholars have therefore argued that code generation creates both opportunities for individualized scaffolding and challenges for assessment, foundational skill development, and academic integrity (Becker et al., 2023; Denny et al., 2024; Finnie-Ansley et al., 2022). Interviews with instructors indicate that simple prohibition is unlikely to be sustainable and that curricula will need to make AI-supported work and its evaluation explicit (Lau & Guo, 2023).

The learner’s level of expertise does not solve the problem automatically. Prather et al. (2024) observed that novice programmers experienced cognitive and metacognitive difficulties when interacting with Copilot, including uncertainty about whether suggestions were appropriate and how to recover when the tool disrupted their plan. At the same time, professional expertise is not a guarantee against adoption: an experiment using an objective code-similarity measure found that explanatory comments increased uptake of AI-generated JavaScript code regardless of participants’ reported development expertise (C. Li et al., 2026). These findings shift attention from whether an AI can produce a solution to how users determine the evidential threshold for accepting it.

### 2.2. Risks in GAI-Assisted Coding

Generated code poses at least four linked risks. The first is functional error. Benchmark studies show that AI assistants can solve many conventional problems but also produce buggy or non-reproducible solutions, particularly as tasks require coordination across methods or richer context (Moradi Dakhel et al., 2023). The second is security. Pearce et al. (2022) demonstrated that Copilot suggestions can contain security weaknesses, while Perry et al. (2023) found that AI assistance can lead users to produce more insecure code and can increase confidence in insecure solutions. The third is maintainability and contextual fit: a locally functioning answer may conflict with project architecture, hidden requirements, style constraints, or future changes. The fourth is epistemic opacity. When a learner cannot explain the generated code, execution feedback may correct some defects without repairing the learner’s mental model.

These risks expose the weakness of an execution-only criterion. Unit tests sample specified behaviors and are valuable, but passing them does not establish the absence of untested defects, security vulnerabilities, or misunderstood assumptions. Professional code review consequently centers on change understanding as well as defect detection (Bacchelli & Bird, 2013), and large-scale industrial practice frames review as a social and educational process serving norm maintenance and knowledge transfer rather than as a final execution check (Sadowski et al., 2018). GenAI adds a distinctive asymmetry: the system can generate more code, and more alternative solutions, than a learner can carefully inspect. Oertel et al. (2025) found that checking several Copilot alternatives can improve the chance of encountering a correct solution, but exhaustive checking is rarely cost-effective. Verification is thus an allocation decision under constraints, not a binary moral trait.

### 2.3. Verification, Appropriate Reliance, and Trust Calibration

Research on human-automation interaction provides a useful starting point. Trust is an attitude that helps users decide whether to rely on automation under uncertainty, but well-designed human-automation systems require appropriate rather than maximal trust (Lee & See, 2004). Trust varies with dispositional, situational, and learned factors (Hoff & Bashir, 2015). More importantly, reliance is a behavior, and it may diverge from stated trust. The concept of appropriate reliance therefore evaluates whether a person accepts correct AI advice and rejects incorrect advice at the case level (Schemmer et al., 2023).

Automation complacency and automation bias explain why plausible output can suppress checking. Complacency reflects insufficient monitoring when attention is allocated elsewhere, whereas automation bias produces omission and commission errors when users defer to an automated aid. Both can affect novices and experts and are not eliminated by simple warnings or practice (Parasuraman & Manzey, 2010). In AI-assisted decision making, explanations alone do not consistently solve overreliance. Cognitive forcing interventions can reduce acceptance of incorrect advice, but the interventions users like least may be the ones that induce the most deliberation (Buçinca et al., 2021). Verification also depends on effort: explanations reduce overreliance when they meaningfully lower the cost of checking relative to the cost of solving the task independently (Vasconcelos et al., 2023). These findings are directly relevant to code generation, where compiling, testing, tracing, and inspecting code vary greatly in cognitive and temporal cost.

An epistemic-vigilance perspective adds a second layer. Communication is useful because it transfers information, but it also exposes recipients to error and misinformation; epistemic vigilance comprises mechanisms for evaluating the source and the content of communicated claims (Sperber et al., 2010). AI-generated code is a communicated claim in executable form. Source-level cues include beliefs about the model, tool, provider, and prior reliability. Content-level cues include consistency with requirements, line-by-line intelligibility, tests, documentation, and comparison with independent knowledge. A student who treats successful execution as sufficient privileges one content cue and closes monitoring early. A student who demands an explanation, contrasts alternatives, or consults documentation uses a broader evidential set. Trust calibration and epistemic vigilance thus converge on the same educational problem: how learners set and update the threshold for accepting AI-produced code.

### 2.4. Metacognition, Self-Regulated Learning, and Cognitive Offloading

Metacognition encompasses knowledge of one’s cognition and its monitoring (Flavell, 1979), together with the regulation of that cognition, that is, deciding what to do next on the basis of what one takes oneself to understand (Brown, 1987). Self-regulated learning (SRL) extends this logic across forethought, performance, monitoring, strategy selection, and reflection (Panadero, 2017; Zimmerman, 2002). Programming is particularly metacognitive because successful problem solving requires learners to represent the task, choose a strategy, monitor progress, diagnose impasses, and evaluate a solution. Explicit guidance that prompts programmers to articulate and monitor problem-solving steps can improve both process awareness and performance (Loksa et al., 2016).

GenAI introduces offloading into this cycle. Cognitive offloading uses external action or resources to reduce internal information-processing demands (Risko & Gilbert, 2016). Offloading is not intrinsically harmful: external tools can preserve limited cognitive resources for planning, abstraction, and evaluation, and in a survey of university students, GenAI features were associated with lower perceived cognitive load (Rehman et al., 2026). Yet decisions to offload are themselves metacognitive and can be based on inaccurate judgments of internal ability or external reliability (Risko & Dunn, 2015). Recent work on GenAI further distinguishes useful offloading from concern that the product or competence no longer feels cognitively owned (Su et al., 2026). For programming students, the central question is therefore not whether offloading occurs, but whether monitoring and control remain aligned with the learning purpose and risk of the task.

Generic SRL and code-specific verification need not coincide. A highly regulated student may set efficient goals, improve prompts, divide work among tools, and reflect on task completion while still performing little source-level review. Conversely, a learner with modest general study routines may carefully verify a high-stakes algorithm because failure is salient. This distinction is reinforced by longstanding measurement concerns: broad self-report questionnaires capture perceived tendencies, whereas online traces and task-specific measures capture enacted regulation more directly (Panadero, 2017; Veenman et al., 2006). The present study therefore treats generic metacognitive self-regulation as one potential resource, not as a synonym for verification.

### 2.5. Existing Literature Gaps

Five gaps follow from this literature. First, construct coverage is incomplete. GenAI studies commonly measure acceptance, intention, frequency, trust, usefulness, or broad literacy, but rarely isolate the belief that successful execution constitutes adequate verification. Without this distinction, an attitude toward AI can be mistaken for the decision rule that actually terminates checking.

Second, measurement often relies on a single source and a single level of abstraction. Capability benchmarks evaluate generated code without observing users; laboratory studies observe bounded tasks; surveys capture generalized perceptions; and qualitative studies reveal strategies without estimating their population-level associations. Self-reported trust is also frequently treated as a behavioral proxy even though appropriate reliance is case-specific. A stronger design should separate latent beliefs from reported behavior, assess the psychometric quality of any new measure, and acknowledge that reported frequency is not equivalent to logged conduct or verified performance.

Third, participant coverage remains uneven. Research has focused on introductory novices, instructors, crowd participants, and professional developers. Computer-science undergraduates in the middle and later stages of a degree occupy a different position: they possess some disciplinary knowledge, use GenAI across diverse tasks, and are close to entering AI-mediated software work. Chinese students are especially underrepresented in the empirical literature despite rapid uptake of domestic and international GenAI tools.

Fourth, quantitative studies of AI use often report a single model selected from many possible specifications and rely on self-report scales that are vulnerable to response styles such as acquiescence. Data-driven model selection capitalizes on chance (MacCallum et al., 1992), and agreement tendencies can distort the correlations between positively and negatively worded constructs (Billiet & McClendon, 2000; Podsakoff et al., 2012). Conclusions are more credible when they are shown to hold across the reasonable alternatives that an analyst could have chosen (Steegen et al., 2016).

Fifth, mixed-methods integration is often deferred to parallel summaries. A convergent design should specify whether qualitative evidence confirms, qualifies, conflicts with, or uniquely extends quantitative findings (Fetters et al., 2013). Joint displays make those relationships visible and prevent interview quotations from serving merely as illustrations of significant coefficients (Guetterman et al., 2015). The present study addresses these gaps by linking psychometrically evaluated beliefs, robustness-tested associations, and students’ own accounts of how they decide that checking is sufficient.

## 3. Theoretical Framework

This study conceptualizes verification of AI-generated code through a metacognitive monitoring-and-control perspective. Metacognitive and self-regulated learning models distinguish between monitoring one’s task, knowledge, and performance and using those appraisals to regulate subsequent action (Zimmerman, 2002; Panadero, 2017). Applied to AI-generated code, verification involves a judgment about when the available evidence is sufficient to accept, revise, or reject generated output.

Verification is defined here as checking whether a specific AI-generated output is correct and fit for purpose before it is accepted. It can be carried out by the learner, for example by tracing or explaining the code line by line or by testing it against cases the learner has constructed, or it can be delegated to another tool. Two further practices are related to verification but are not instances of it. Independent practice without AI maintains the unaided competence on which later checking depends, but it does not evaluate any particular output. Anticipatory non-delegation is a decision, taken before any code is generated, not to use AI for tasks judged to be error-prone. The three practices share a function, preserving epistemic control over AI-assisted work, yet they differ in timing, object, and purpose, and a student may engage in one without the others. They are therefore not interchangeable manifestations of a single reflective construct (Bollen & Lennox, 1991) and are examined as separate outcomes. Of the three, only line-by-line comprehension before use refers directly to the verification of AI-generated code.

The framework therefore distinguishes between broader appraisals and a more proximal belief about verification. Perceived usefulness and trust concern how valuable and reliable the AI system is judged to be, but appropriate-reliance research shows that trust does not necessarily imply uncritical acceptance (Lee & See, 2004; Schemmer et al., 2023). Perceived AI literacy reflects learners’ perceived capacity to use and evaluate AI, whereas generic self-regulation reflects broader planning, monitoring, and strategic regulation of learning (Panadero, 2017). Preference for immediate payoff may be associated with less checking because successful execution provides salient feedback while continued inspection imposes additional effort, an account consistent with automation complacency and cognitive offloading (Parasuraman & Manzey, 2010; Risko & Gilbert, 2016).

At the center of the framework is verification-dispensability belief (VDB), defined as the endorsement of reasons for treating further checking, or the unaided practice on which checking depends, as unnecessary. Its most direct form is the view that code which passes its tests can be merged without further examination; related forms rest on confidence that models are usually correct or that manual coding skill is no longer needed. This code-specific belief is expected to be more proximally related to reported verification-related practices than broader dispositions such as trust, perceived AI literacy, or generic self-regulation. Because the model relating these constructs was identified through exploratory analysis of the same data, the associations are examined descriptively, and their stability is evaluated across alternative analytic choices.

## 4. Method

### 4.1. Research Design

The study used a convergent parallel mixed-methods design. Survey and interview data were collected during the same period in July 2026, analyzed separately, and integrated after strand-specific results had been established. The quantitative strand addressed RQ1 through descriptive statistics and group comparisons and RQ2 through an exploratory structural equation model with robustness analyses. The qualitative strand addressed RQ3 through thematic analysis using an inductively developed codebook. Integration used a joint display that classified the relationship between each qualitative theme and the corresponding survey result as confirmation, qualification, conflict, or extension (Fetters et al., 2013; Guetterman et al., 2015).

### 4.2. Setting, Participants, and Procedure

The online survey was administered to students in computer-related majors at a comprehensive university in China. The available recruitment record does not document probability sampling; the sample was therefore treated as a convenience sample. All respondents provided online informed consent. Of 372 submitted questionnaires, 78 unique cases were excluded following recommendations for detecting careless responding (Meade & Craig, 2012). Six respondents had entire scale blocks missing after reporting that they had never used GenAI, 61 gave the same numeric response throughout the Likert blocks, and 72 had within-person response variation below 0.20; these criteria overlapped, so their counts do not sum to 78. The final quantitative sample comprised 294 students.

The analytic sample included 201 men (68.4%) and 93 women (31.6%). The largest majors were computer science and technology (*n* = 124, 42.2%), software engineering (*n* = 84, 28.6%), and intelligent science and technology (*n* = 59, 20.1%). Most respondents were third-year undergraduates (*n* = 196, 66.7%); 43 (14.6%) were master’s students. A total of 184 students (62.6%) used GenAI almost daily or several times a day, and 173 (58.9%) had paid for a GenAI service at least occasionally. Because recruitment relied on convenience sampling at a single institution and two-thirds of respondents were third-year undergraduates, the sample represents computer-science students in this institutional context rather than Chinese computer-science students in general.

Eleven students (10 men, 1 woman) from computer-related programs at the same university took part in semi-structured interviews: one first-year, one second-year, and seven third-year undergraduates, one master’s student, and one doctoral student. Participants were recruited from survey respondents who indicated willingness to take part in a paid online interview and received 30–60 yuan for their time. Interviews were conducted in Mandarin through Tencent Meeting between 29 and 31 July 2026 by a trained graduate research assistant who had no teaching, assessment, or administrative relationship with the participants. They lasted 22 to 53 min (*M* = 38.9). The interview guide covered tool histories; AI use in coursework, projects, competitions, and internships; circumstances for independent versus AI-assisted coding; experiences with hidden defects; whether generated code was fully understood; personal thresholds for checking; teacher and peer norms; perceived skill change; and the future professional role of code review. Interviews were audio-recorded with separate consent and transcribed verbatim in Chinese. Reporting of the interview procedure follows the relevant items of the COREQ checklist (Tong et al., 2007).

The adequacy of the interview sample was judged against the information-power criteria of Malterud et al. (2016). The aim was narrow (decisions about checking AI-generated code), all participants had direct experience of the practice, the analysis was guided by an explicit codebook, and the interviews were extended dialogues averaging almost 40 min. Code saturation was additionally monitored in the order in which interviews were conducted (Hennink et al., 2017; Section 5.6).

The study was approved by the Institutional Review Board of Qingdao University (approval no. QDU-PSY-20260603A). Participation was voluntary, and survey consent and interview recording consent were obtained separately.

### 4.3. Measures

All attitudinal items used seven-point agreement scales (1 = strongly disagree, 7 = strongly agree), with higher scores indicating stronger endorsement. The questionnaire was administered in Chinese; the Chinese wording and English translation of every item analyzed in this study are provided in Section S3 of the Supplementary Materials.

Three measures were adopted from Jiang and Wang (2026). Preference for immediate payoff (IMP; two items) assessed preference for rapid results and impatience with delayed rewards. Trust in AI (TRU; two items) assessed confidence in the usability of AI output and willingness to act on AI advice. Perceived AI literacy (AIL; three items; LIT in the original study) assessed effective prompting, evaluation of AI output, and selection of appropriate AI tools. The validated two-item form of IMP has also been used by Su et al. (2026).

Two additional questionnaire items were excluded before substantive analysis because they did not belong to the intended constructs. A reverse-worded IMP item reduced internal consistency substantially (α = 0.043 versus 0.815 for the validated two-item form), consistent with the item-removal decision in Jiang and Wang (2026) and evidence that reverse wording can introduce method effects (van Sonderen et al., 2013). An additional TRU item concerned scrutinizing and revising AI output and showed stronger conceptual and empirical overlap with critical evaluation (HTMT = 0.872).

Four study-specific attitudinal measures were used. Verification-dispensability belief (VDB; three items) was measured with statements that merging AI-written code without line-by-line examination is acceptable once all tests pass (VDB1), that there is little need to practice writing code by hand now that code is completed by AI (VDB2), and that understanding how large models work makes routine verification unnecessary because the models are usually correct (VDB3). Only VDB1 refers directly to execution evidence; the label therefore names the content common to the three items, namely reasons for dispensing with further checking or with the practice that sustains it. Verification commitment (VC; three items) assessed refusal to submit code that could not be explained, writing an independent version before comparing it with AI output, and verifying error-prone task types before use. The VDB and VC items alternated within a single block with opposite keying, which allowed acquiescence to be modeled. Metacognitive self-regulation (SRL; three items) assessed planning, independent exploration, and post-task reflection, and perceived benefit (BEN; three items) assessed the perceived efficiency and value of GenAI for programming-related learning. Psychometric properties of these measures were evaluated in the present sample (Boateng et al., 2018; Clark & Watson, 1995); independent validation is not yet available.

The three verification-related practices were measured with single items referring to the previous month and were analyzed as separate outcomes: the proportion of AI-generated code that the student had understood line by line before using it (line-by-line comprehension), completion of programming or algorithm exercises without AI (independent practice), and giving up AI use in favor of working unaided or consulting documentation because AI was expected to be error-prone for the task (anticipatory non-delegation). All three used five ordered response categories labeled almost none, less than half, about half, more than half, and almost all. Although the second and third stems asked about the number of occasions, these proportional labels mean that responses indicate relative rather than absolute frequency. All three are retrospective self-reports and do not measure observed checking, the quality of checking, or code correctness.

Delegation breadth (DELEG) was the number of nine programming-related domains in which respondents reported using GenAI, including explanation, code generation, debugging, algorithms, project development, data processing, technical writing, environment configuration, and preparation for competitions, examinations, or employment.

Seven further scales from the broader questionnaire that has been administered on a larger scale (self-efficacy, AI-related anxiety, fear of missing out on AI, critical evaluation of AI output, peer norms, perceived accessibility and anthropomorphism, and AI dependency) were not part of the substantive model. They were used only in the descriptive group comparisons and as covariates in robustness analyses; their items appear in Section S3.

### 4.4. Quantitative Analysis

#### 4.4.1. Descriptive Analysis, Group Comparisons, and Measurement Model

RQ1 was examined using frequencies, means, correlations, and one-way ANOVA across narrow (≤3 domains), medium (4–5), and broad (≥6) delegation groups. Gender differences were tested with Welch *t* tests and student-level differences with one-way ANOVA, with Benjamini–Hochberg correction across each family of 18 comparisons (Benjamini & Hochberg, 1995). Equivalence of the three practices across gender was evaluated with two one-sided tests using bounds of *d* = ±0.35, the smallest effect detectable with 80% power in this sample. Measurement models were estimated in R 4.5.2 with lavaan 0.6.21 and semTools 0.5.8. A seven-factor confirmatory factor analysis (CFA) was fitted with robust maximum likelihood (MLR), and construct quality was evaluated using standardized loadings, coefficient omega, average variance extracted (AVE), heterotrait–monotrait (HTMT) ratios, and the Fornell–Larcker criterion (Fornell & Larcker, 1981; Henseler et al., 2015; Hu & Bentler, 1999).

Two measurement questions were examined before substantive modeling. First, to test whether the three practices could be treated as indicators of one reflective factor, a model in which they loaded on a latent verification factor was compared with a model in which they were separate, correlated outcomes. The latent-factor model constrains the associations of every predictor with the three practices to be proportional, so a significant scaled chi-square difference indicates that the practices are not interchangeable indicators (Bollen & Lennox, 1991; Rhemtulla et al., 2020). Second, because the VDB and VC items were oppositely keyed, a random-intercept factor with equal loadings on all six items was added to capture individual differences in acquiescent responding (Billiet & McClendon, 2000; Maydeu-Olivares & Coffman, 2006). Residual variances estimated as negative but not significantly different from zero were fixed at zero.

#### 4.4.2. Exploratory Structural Model

RQ2 was addressed with a structural model whose form was identified through an exploratory specification search. In the initial analysis, 216 candidate models were screened: 63 single-mediation, 29 chain-mediation, 40 moderation, and 84 moderated-mediation models. Of these, 76 (35.2%) yielded *p* < .05, and 30 mediation tests remained significant after Benjamini–Hochberg correction; none of the 124 interaction tests did (smallest *q* = 0.059; Table S13). The retained model was therefore additive. Specification searches are an established exploratory use of structural equation modeling, provided that the search is reported in full, multiplicity is controlled, and the retained model is checked against capitalization on chance (Cudeck & Browne, 1983; MacCallum & Austin, 2000; Marcoulides et al., 1998). Because the structure was chosen after inspecting the data, a procedure that capitalizes on chance (MacCallum et al., 1992), the model is labeled exploratory throughout, following the distinction between hypothesis-generating and hypothesis-testing analyses (Nosek et al., 2018; Wagenmakers et al., 2012); its paths are interpreted as cross-sectional associations, and indirect effects are reported only as decompositions of covariances, not as evidence of mediating processes (Maxwell & Cole, 2007; Rohrer, 2018). The term specification search refers to this post hoc search over candidate structures rather than to exploratory structural equation modeling in the sense of Asparouhov and Muthén (2009).

The model specified perceived benefit (BEN), the counterpart of performance expectancy in technology-acceptance research (Venkatesh et al., 2012), as a predictor of trust (TRU); TRU, immediate-payoff preference (IMP), perceived AI literacy (AIL), and self-regulation (SRL) as predictors of VDB; and VDB, BEN, IMP, AIL, and SRL as predictors of each of the three practices, whose residuals were allowed to correlate. The model was estimated with MLR, and bias-corrected 95% confidence intervals for all paths and for the indirect associations IMP → VDB → practice and BEN → TRU → VDB → practice were obtained from 5000 bootstrap samples (Preacher & Hayes, 2008). The model was re-estimated independently in Mplus 8.3.

#### 4.4.3. Robustness Analyses

Because the model was not specified in advance, the stability of each association was examined across six sets of alternative analytic choices. (a) The model was re-estimated with WLSMV, treating all indicators as ordered categories (C.-H. Li, 2016); the loadings of the two immediate-payoff items were constrained to be equal, which prevented an improper negative residual variance. (b) VDB was replaced by its single execution-referenced item, VDB1. (c) Acquiescence was controlled by adding the random-intercept factor to a latent regression of the three practices on VDB, AIL, SRL, TRU, IMP, and BEN. (d) The model was re-estimated in 100 random splits of the sample (200 half-samples). (e) In a multiverse analysis (Simonsohn et al., 2020; Steegen et al., 2016), each association between a focal predictor and a practice was estimated under four screening rules (the main rule; all 366 complete responses; additionally excluding respondents who finished in less than half the median completion time; and additionally excluding respondents who agreed strongly with both statements of a near-contradictory VDB–VC pair), four covariate sets (none; the model predictors; the model predictors plus VC; and all 18 measured variables), and two estimators (ordinary least squares and ordinal logistic regression), giving 32 specifications per association. VDB was additionally scored as VDB1 alone and without VDB2, whose content overlaps with independent practice. (f) Out-of-sample predictive accuracy was estimated with 20 repetitions of 10-fold cross-validation (Yarkoni & Westfall, 2017).

An association was described as consistent when it had the same sign in all robustness analyses and was significant in the main model, in every alternative estimation, and in at least 90% of multiverse specifications; other associations were described as tentative. These criteria were set after the initial analyses and are therefore descriptive conventions rather than confirmatory thresholds.

### 4.5. Qualitative Analysis and Mixed-Methods Integration

Interview transcripts were analyzed with thematic analysis using a codebook developed inductively from the transcripts (Braun & Clarke, 2006, 2021). Initial coding focused on decision points in checking AI-generated code: when students delegated tasks, what counted as sufficient understanding, what prompted deeper checking, how errors were handled, and how teachers or peers shaped acceptable practice. Candidate themes were compared within and across participants and refined into a written manual specifying, for each theme, a definition, inclusion and exclusion criteria, discrimination rules for easily confused themes, and a priority rule for units meeting more than one definition (Section S2). Negative and dissonant cases were retained.

Coding units were paragraphs of respondent speech that contained at least 25 Chinese characters and at least one of 28 terms referring to AI, code, checking, understanding, coursework, or institutional rules (Section S1). This rule was adopted as a focused strategy, because the analysis concerned how students talked about checking and delegating code rather than their full accounts of AI use. It retained 389 of 511 respondent paragraphs. Of the 122 excluded paragraphs, 85 were shorter than 25 characters, 29 were longer but contained none of the terms, and 8 were formulaic closings or similar; together they accounted for 9.4% of respondent text (Table S14). The 29 longer excluded paragraphs were re-examined against the codebook; none described checking AI-generated code or the allocation of such checking (they concerned tool inventories, study backgrounds, career plans, and questions about the interview), and all would have received the residual code.

All 389 units were coded by the first author using the manual, after training by the third author, who then reviewed every code assignment against the manual; disagreements were identified, highlighted and resolved by discussion among all authors, with the corresponding author adjudicating when agreement could not be reached. Because the analysis did not apply blind independent coding between any two authors, Cohen’s kappa could not be computed or reported.

The research team comprised education researchers who study learners’ use of GenAI, none of whom taught the participants. Because the team’s prior work had examined dependence on GenAI, there was a risk of foregrounding risks over benefits. To counter this risk, the codebook included themes describing competent and deliberate delegation, dissonant cases were retained, and the joint display recorded conflicts between the strands as well as convergences (Olmos-Vega et al., 2023). Interview quotations were translated into English by the third author and checked for meaning against the Chinese originals by the corresponding author who has over 8 years of studying experience in the United States; the Chinese originals are given in Table S16 so that readers can assess the translations (Temple & Young, 2004).

Integration occurred after both strands had been analyzed. For each survey result that bore on a qualitative theme, the relationship was classified as confirmation (both strands point to the same conclusion), qualification (the interviews limit or specify the conditions under which the survey result holds), conflict (the interviews indicate that the survey result is incomplete or misleading, including where the survey did not measure a practice that students described), or extension (the interviews add a dimension absent from the survey) (Fetters et al., 2013).

## 5. Results

### 5.1. Measurement Quality and Preliminary Diagnostics

The seven-factor CFA fitted the data well, χ^2^(131) = 208.08, *p* < .001, CFI = 0.979, TLI = 0.972, RMSEA = 0.051, 90% CI [0.038, 0.064], SRMR = 0.042. Standardized loadings ranged from 0.635 to 0.972, coefficient omega from 0.780 to 0.972, and AVE from 0.544 to 0.920. HTMT ratios were ≤0.750, and all constructs satisfied the Fornell–Larcker criterion (Tables S1–S4). Table 1 reports the measurement properties of the focal constructs.

Two further measurement results informed the substantive analyses (Table S7). First, the model treating the three practices as indicators of one latent factor fitted significantly worse than the model treating them as separate correlated outcomes, scaled Δχ^2^(10) = 58.56, *p* < .001 (WLSMV with all indicators ordered: Δχ^2^(10) = 54.81, *p* < .001; AIC = 13,125 vs. 13,099; BIC = 13,324 vs. 13,335). The proportionality constraints implied by a reflective factor were therefore rejected, and the practices were analyzed separately; their intercorrelations ranged from 0.36 to 0.56. Second, the six oppositely keyed VDB and VC items showed a pattern characteristic of acquiescence. In the two-factor model, VDB and VC were positively correlated (*r* = 0.443) although their content is opposed, and 50 respondents (17.0%) agreed or strongly agreed with both statements of at least one near-contradictory pair. Adding a random-intercept factor, with the residual variance of one VC item fixed at zero because its estimate was negative and not significantly different from zero, improved fit (CFI = 0.986 vs. 0.945; RMSEA = 0.064 vs. 0.128; BIC = 5446 vs. 5476); the variance of the acquiescence factor was significant (0.65, *p* < .001), and the VDB–VC correlation became negative (*r* = −0.336, *p* = .014). The reverse-worded IMP item and the reverse-combined six-item verification score (α = 0.337) had already shown poor internal consistency, and VDB and VC were retained as separate constructs.

### 5.2. RQ1: Delegation Breadth and Reported Verification-Related Practices

Students reported using GenAI in an average of 5.70 of nine programming-related domains (*SD* = 2.82), with 59.2% using it in at least five. Use was most common for concept explanation and code examples (91.2%), code generation or completion (81.0%), debugging and error diagnosis (78.9%), and algorithms or data structures (68.4%).

Verification-related practices were reported less often. Mean line-by-line comprehension before use was 2.88 (*SD* = 1.17), independent practice without AI 2.32 (*SD* = 1.01), and anticipatory non-delegation 2.40 (*SD* = 0.98), on scales from 1 (almost none) to 5 (almost all).

Delegation breadth was unrelated to line-by-line comprehension, *r* = 0.045, *p* = .441, to independent practice, *r* = −0.088, *p* = .134, and to anticipatory non-delegation, *r* = −0.053, *p* = .363. Line-by-line comprehension did not differ across narrow, medium, and broad delegation groups, *F*(2, 291) = 0.69, *p* = .504. Figure 1 displays the breadth of delegation alongside the distribution of each practice.

### 5.3. RQ1: Gender and Student-Level Differences

No gender difference survived Benjamini–Hochberg correction across the 18 variables (smallest *q* = 0.076, for AI-related anxiety). The three practices were statistically equivalent across gender within bounds of *d* = ±0.35: line-by-line comprehension, *d* = −0.09, equivalence *p* = .020; independent practice, *d* = 0.07, *p* = .013; and anticipatory non-delegation, *d* = 0.11, *p* = .024 (Table S5).

Student level was associated with two practices after correction. Line-by-line comprehension differed across levels, *F*(2, 288) = 8.35, *p* < .001, η^2^ = 0.055, *q* = 0.005, following a non-monotonic pattern: first- and second-year students reported the highest level (*M* = 3.48), third-year and later undergraduates the lowest (*M* = 2.71), and postgraduates an intermediate level (*M* = 3.17). Independent practice declined across levels, *F*(2, 288) = 6.04, *p* = .003, η^2^ = 0.040, *q* = 0.024, from 2.85 among first- and second-year students to 2.30 among later undergraduates and 2.08 among postgraduates. That the same grouping variable showed different patterns for the two practices is a further indication that they are not interchangeable. No other student-level difference survived correction. Because the data are cross-sectional and the first- and second-year group was small (*n* = 33), these differences are cohort patterns rather than evidence of developmental change. Table 2 reports the comparisons for the model variables and practices.

### 5.4. RQ2: Exploratory Structural Model with Separate Practices

The structural model with separate outcomes fitted the data acceptably, χ^2^(126) = 223.49, *p* < .001, CFI = 0.974, TLI = 0.965, RMSEA = 0.057, 90% CI [0.045, 0.069], SRMR = 0.052; Mplus reproduced the chi-square value and all standardized estimates (Table S6). The model accounted for 56.1% of the variance in trust, 32.5% in VDB, and 27.0%, 11.1%, and 6.9% in line-by-line comprehension, independent practice, and anticipatory non-delegation, respectively.

Perceived benefit was strongly associated with trust (*β* = 0.749, *p* < .001), but trust was not associated with VDB when the other predictors were included (*β* = 0.105, *p* = .292), and the indirect association BEN → TRU → VDB was not significant for any practice. Immediate-payoff preference was positively associated with VDB (*β* = 0.533, *p* < .001), whereas perceived AI literacy (*β* = 0.002) and self-regulation (*β* = −0.103) were not.

VDB was negatively associated with all three practices: line-by-line comprehension (*β* = −0.408, *p* < .001), independent practice (*β* = −0.301, *p* = .001), and anticipatory non-delegation (*β* = −0.245, *p* = .007). Perceived AI literacy was positively associated with line-by-line comprehension (*β* = 0.353, *p* = .002) but not with independent practice (*β* = 0.207, *p* = .147) or anticipatory non-delegation (*β* = 0.221, *p* = .092). Self-regulation, perceived benefit, and immediate-payoff preference had no direct associations with any practice. The covariance between immediate-payoff preference and each practice was statistically accounted for by VDB (indirect *β* = −0.218, −0.160, and −0.130; all bias-corrected 95% CIs excluded zero), but because the data are cross-sectional, this decomposition does not establish that immediate-payoff preference shapes VDB or that VDB shapes practice (Maxwell & Cole, 2007). Table 3 reports all paths, and Figure 2 presents the model.

### 5.5. RQ2: Robustness of the Associations

Table 4 summarizes the stability of the associations across the robustness analyses. The negative associations between VDB and the three practices had the same sign in every analysis. They remained significant when all indicators were treated as ordered (*β* = −0.485, −0.328, and −0.279), when VDB was represented only by its execution-referenced item (*β* = −0.395, −0.237, and −0.180), and when acquiescence was modeled together with all focal covariates (*β* = −0.681, −0.831, and −0.469; all *p* ≤ .004). In the multiverse analysis, the associations of VDB with line-by-line comprehension and independent practice were significant in all 32 specifications and the association with anticipatory non-delegation in 93.8%; results were unchanged when VDB was scored without VDB2 (Table S11). All three VDB associations therefore met the criteria for consistency, although the association with anticipatory non-delegation was significant in only 54.3% of half-samples, compared with 94.0% for line-by-line comprehension and 73.4% for independent practice.

Perceived AI literacy was positively associated with line-by-line comprehension in every analysis (all 32 multiverse specifications; 66.3% of half-samples), but its associations with independent practice and anticipatory non-delegation were significant in only 40.6% and 37.5% of specifications and 17.1% and 24.1% of half-samples; these associations are tentative. Self-regulation was not significantly associated with any practice in the main or alternative models, and its multiverse estimates varied in sign. The association of trust with VDB depended on the estimator: it was non-significant under MLR but significant under WLSMV (*β* = 0.229), so the corresponding indirect association is not interpreted. The positive associations of verification commitment with the practices, which appeared when VDB was controlled (e.g., *β* = 0.381 for line-by-line comprehension), became non-significant once acquiescence was modeled (*β* = −0.201, −0.070, and −0.107), which indicates a response-style suppression effect rather than a substantive protective association (Tables S8–S10).

Cross-validation indicated modest out-of-sample prediction. For line-by-line comprehension, the model predictors explained 21.4% of the variance out of sample, compared with 11.3% when VDB was omitted. For independent practice and anticipatory non-delegation, out-of-sample *R*^2^ values were 3.8% and 1.9%, and a model with all 18 measured variables predicted anticipatory non-delegation no better than the sample mean (*R*^2^ = −0.014), which indicates that the larger model overfitted the data (Table S12).

### 5.6. RQ3: Students’ Accounts of Checking AI-Generated Code

All nine themes had appeared by the third interview, and no new theme emerged in the remaining eight (Table S15), which indicates code saturation for this focused analysis (Hennink et al., 2017). Of the 389 coded units, 137 received one of the nine thematic codes, 248 bore on none of them, and four were left unassigned. Purpose-based allocation of checking was the most widespread theme (34 units; 11 participants), followed by delegated verification (30 units; 10 participants), institutional legitimation (21 units; 9 participants), and verification as a future professional role (16 units; 9 participants). Checking actually performed by the student was described in 8 units by 5 participants. These counts describe how often themes were voiced in the interviews, not how common the practices are.

Checking was allocated by task purpose. Students distinguished tasks intended for learning from those oriented toward producing a usable output. One third-year student explained: “For algorithm problems or large course assignments, such as the data-structures course project, I basically hand-code everything and have not used AI at all. AI comes to mind first for larger projects such as competitions, especially when time is tight” (03). Another drew the same boundary between competition and front-end work: “For algorithm-competition code, I always fully understand all of the logic before submitting … For things I am not good at, such as front-end work, I do not dig into the underlying logic” (07). In low-stakes tasks, a running program could be treated as enough: “I only need to check whether the final code runs properly” (10).

Understanding was not always defined line by line. Several participants treated overall logic, execution flow, data flow, or the function of each component as sufficient: “There is no need to reproduce the code completely on my own without AI, but I do need to understand clearly what each part of the code does” (11). By this standard, low scores on line-by-line comprehension did not necessarily indicate that students regarded their understanding as inadequate.

Checking was frequently delegated to AI. Students described asking the same model to inspect its own output, submitting finished programs for a second review, or chaining models: “We now also have a loop workflow: first generate the code with GPT, then use Claude Code to check whether the code is right or wrong” (04). Another student accepted code only after an AI review: “only after the review is passed do I confirm that the code is usable” (09). The survey item asked only about comprehension achieved by the student and therefore did not capture this practice.

Checking that students had carried out themselves was described far less often. Eight units from five participants recounted such checking: reviewing each change before committing it, going through a whole file before submission, running tests and then inspecting the affected modules, or rolling back and rewriting when a fault could not be located. One student described detecting a defect that execution had concealed: “The final output of the code was correct, but its internal logic was wrong … I can detect this kind of problem when I go through the code in full” (05). Thirty units from ten participants described checking executed by a model, against eight units from five participants describing checking done by the student. Both counts are lower bounds, because the interview guide asked about neither practice directly and Chinese habitual markers do not reliably distinguish a routine the speaker performs from a standard the speaker endorses.

Self-regulation could serve efficient delegation as well as checking. A student who described a disciplined habit of attempting problems before consulting AI also attributed failures of AI-written code to the user: “If code written by AI does not run now, you have to look for the cause in yourself … AI is very good at writing code now” (02).

Institutional messages shaped acceptable levels of checking. Participants described courses that required AI generation, teachers who emphasized tool use over code understanding, and formal restrictions that coexisted with widespread use: “The teacher said that whatever is generated with tokens is of guaranteed quality, so I generated everything with Claude Code” (02); “On the surface, the teacher explicitly prohibits AI in assignments, but in practice almost all students use AI when writing the C-language course project” (07).

Three further themes concerned students’ awareness of an illusion of competence, their expectation that programmers would increasingly act as reviewers of AI-generated code, and their distinction between declining production skill and retained judgment. One student named the illusion directly: “Many people, including me, have the illusion that once AI knows it, I know it … AI has basically taken away our ability to judge whether code is correct” (01). Others described review as the future core of the profession, “this ability to screen and review will become a core competitive advantage” (11), or separated what was declining from what was not: “my hands-on ability to write code by hand has indeed declined. But my problem analysis, top-level design, and thinking for solving complex problems have kept improving” (04). Chinese originals of all quotations are given in Table S16.

### 5.7. RQ3: Mixed-Methods Integration

Table 5 classifies the relationship between each qualitative theme and the corresponding survey result. The interviews confirmed three survey results, qualified three, conflicted with one, and extended one. The conflict concerns measurement: students frequently delegated checking to AI and often defined sufficient understanding at the level of process rather than lines of code, whereas the survey measured only line-by-line comprehension achieved by the student. Reported line-by-line comprehension is therefore an incomplete indicator of checking in this population; it underrepresents delegated checking and cannot distinguish thorough from superficial review.

The strands converged most clearly on the role of beliefs about sufficiency. Students who regarded a running program as enough, or who named the illusion that the model’s competence was their own, articulated in their own terms the belief that the survey associated with fewer reported practices. The strands also converged on commitment: interviewees widely endorsed checking as a professional norm, yet accounts of checking actually performed were rare, consistent with the finding that survey endorsements of verification commitment could not be distinguished from acquiescent responding.

## 6. Discussion

This study examined beliefs associated with computer-science students’ reported practices for checking AI-generated code at one Chinese university. Three findings organize the discussion. First, the breadth of GenAI use was unrelated to reported verification-related practices. Second, verification-dispensability belief was the most consistent correlate of fewer reported practices, whereas generic self-regulation showed no association and perceived AI literacy was associated only with line-by-line comprehension. Third, the interviews showed that checking was often allocated by task purpose, defined at the level of process, or delegated to AI, which indicates that the survey captured only part of what students count as verification. Because the survey data are cross-sectional and self-reported and the model was identified exploratorily, these findings are associations that require confirmation with behavioral, longitudinal, or experimental designs.

### 6.1. Reported Verification Was Associated with Beliefs About Sufficiency Rather than with the Amount of AI Use

Students used GenAI across many programming activities, yet delegation breadth was unrelated to all three reported practices. The interviews help explain this decoupling. Students did not appear to occupy a single continuum from independent work to AI dependence; instead, they described shifting between more and less intensive checking according to task purpose. Some preserved unaided work for algorithmic or explicitly learning-oriented tasks while delegating extensively in delivery-focused work. Similar task-dependent shifts between exploratory and acceleration-oriented use have been observed among programmers working with AI coding systems (Barke et al., 2023).

This distinction matters because high AI use should not automatically be interpreted as weak epistemic engagement. The relevant question is not simply whether students delegate, but what evidence they require before accepting delegated output. Interviews further showed that this standard was not always line-by-line comprehension. Some students regarded understanding the overall logic, data flow, or component relationships as sufficient. Such judgments may be defensible in some programming contexts but inadequate in others. Verification therefore needs to be evaluated relative to the purpose and failure costs of the task rather than against a universal requirement for maximal inspection.

The most consistent quantitative result concerned beliefs about when checking can be dispensed with. Students who endorsed merging code once tests pass, or who regarded routine verification or unaided practice as unnecessary, reported fewer verification-related practices, and this association held across estimators, response-style control, screening rules, and covariate sets. The single item referring only to execution evidence showed the same pattern. The pattern is consistent with automation-complacency and cognitive-offloading accounts, in which visible success provides an immediate signal of completion while further inspection requires effort without immediate reward (Parasuraman & Manzey, 2010; Risko & Gilbert, 2016). Immediate-payoff preference was associated with stronger VDB, but decomposing this association into an indirect path in cross-sectional data does not show that preference for immediate results leads students to adopt the belief; the reverse ordering, or a common cause, is equally compatible with the data (Maxwell & Cole, 2007). Generalized trust was not associated with VDB under the main estimator, consistent with the argument that appropriate reliance depends on the evaluation of particular outputs rather than on a global attitude toward the system (Lee & See, 2004; Schemmer et al., 2023).

The findings are therefore compatible with the possibility that some students treat readily available evidence, such as passing tests, as sufficient before further checking would be warranted. Whether such beliefs precede and shape checking, and whether they correspond to what students do when inspecting code, can be established only with task-based and longitudinal evidence.

### 6.2. Generic Self-Regulation Was Not Associated with Reported Verification

Generic metacognitive self-regulation showed no association with any of the three practices in the main model or in any robustness analysis, and it was unrelated to VDB. The interviews suggest why a general capacity for regulation need not translate into checking.

Self-regulation concerns the organization of cognition and behavior toward a goal, but it does not determine what that goal should be (Zimmerman, 2002; Panadero, 2017). A highly self-regulated student may use planning, decomposition, reflection, and strategic tool selection to deepen understanding, but the same capacities can also be used to complete work more efficiently through delegation. In the interviews, students sometimes described disciplined learning routines while simultaneously expressing strong confidence in AI output or minimizing the need for independent checking.

This finding suggests that AI-era metacognition should be specified more precisely. General measures of planning, monitoring, and reflection may be insufficient when the central question concerns whether learners challenge external output. What matters is not only whether learners regulate their activity, but what they regulate toward and what evidence they use to terminate monitoring. Code-specific judgments, confidence calibration, error detection, and explicit verification criteria may therefore be more informative than generic self-regulation alone (Veenman et al., 2006).

The qualitative finding of delegated verification further extends this point. Students frequently asked the same or another model to inspect generated code. Such practices are more deliberate than uncritical acceptance, but they externalize part of the verification process, and the survey item, which referred only to comprehension achieved by the student, did not capture them. Future research should therefore distinguish independent human verification, AI-assisted human verification, AI-delegated verification, and unverified acceptance rather than treating verification as a single continuum.

### 6.3. Perceived AI Literacy Was Associated with Line-by-Line Comprehension Only

Perceived AI literacy showed a narrower pattern. It was associated with line-by-line comprehension in every analysis, but its associations with independent practice and anticipatory non-delegation were inconsistent across specifications and half-samples. Educational studies of AI literacy, trust, and dependency have likewise found these constructs to be intertwined rather than uniformly protective (Wijaya et al., 2024; Zhang et al., 2025). Because the measure captured perceived rather than demonstrated competence, the association may reflect confidence in judging code as much as the ability to do so (Craig et al., 2020).

One interpretation is that students who feel able to evaluate AI output are more likely to report tracing it, whereas maintaining unaided practice and avoiding AI for error-prone tasks depend on other considerations, such as task purpose and time pressure, that the interviews foregrounded. Perceived competence may support scrutiny, but it may also generate confidence that reduces the perceived need for further checking.

The interviews similarly distinguished production skill from diagnostic judgment. Some students reported weaker unaided coding fluency while perceiving improvement in problem framing, architecture, or review. This suggests that AI may redistribute programming expertise rather than simply diminish it. Future work should therefore pair perceived AI literacy with direct diagnostic tasks, such as identifying seeded defects in AI-generated code while reporting confidence. Such designs could distinguish actual competence from calibration and overconfidence.

### 6.4. Implications for Measuring Verification

The integration of the two strands also shows what the interviews added to the survey: they revealed forms of checking that the survey items did not capture and thereby exposed limits in how verification was measured. First, the reported practices were retrospective self-reports, and self-reported metacognitive activity corresponds only partly with observed behavior (Craig et al., 2020; Veenman et al., 2006). Second, the interviews showed that much checking was delegated to other models and that sufficient understanding was often defined at the level of process; a single item about line-by-line comprehension by the student therefore captures only one form of verification. Third, statements endorsing verification were confounded with acquiescent responding, so self-reported commitment was not informative once response style was modeled. Future survey instruments should distinguish human-executed, AI-assisted, and AI-delegated checking, use balanced keying, and be validated against task-based criteria such as the detection of seeded defects, test quality, and objective assessments of code correctness and comprehension.

### 6.5. Theoretical and Practical Implications

The findings suggest a refinement of metacognitive accounts of AI-assisted learning that separates broad regulatory dispositions from domain-specific beliefs about when checking is sufficient. Verification-dispensability belief was not reducible to low self-regulation, low perceived literacy, or high trust, and it was the only construct consistently associated with reported practice. This pattern is consistent with the proposal that code-specific beliefs about sufficiency are more proximal to checking than broad dispositions, a proposal that requires testing against observed checking behavior.

The study also suggests that verification should be treated as situated and partly socially organized. Interviews showed that students adjusted checking to task purpose, redefined what counted as understanding, delegated some verification to AI, and responded to teacher and course norms. Verification thresholds therefore emerge not only from individual cognition but also from assessment requirements and institutional expectations.

For programming education, the implication is not that students should verify every AI-generated line equally. Instead, instruction should make verification criteria explicit. Learning-oriented tasks may require explanation, tracing, or independent reconstruction, whereas delivery-oriented tasks may permit greater delegation but require evidence appropriate to the consequences of error. Students can be asked to justify tests, identify failure cases, explain critical logic, compare alternatives, or document why the available evidence is sufficient. Such practices shift attention from whether AI was used to whether its output was responsibly evaluated.

More broadly, AI literacy should include calibrated judgment rather than prompt proficiency alone. As students increasingly anticipate professional roles centered on reviewing rather than producing routine code, the ability to determine when AI output is trustworthy, what remains uncertain, and what additional evidence is required may become a central component of programming expertise.

### 6.6. Limitations and Future Research

Several limitations qualify the findings. First, all quantitative measures of practice were retrospective self-reports of relative frequency. They are not equivalent to observed checking, to the quality of checking, or to secure code, appropriate reliance, or learning, and they omit checking delegated to AI. Second, the data are cross-sectional; the structural paths and indirect associations describe covariation and do not establish temporal or causal order. Third, the structural model was identified through an exploratory specification search of the same data. The robustness analyses show that the main associations did not depend on particular analytic choices, but they do not substitute for replication in an independent sample. Fourth, VDB, VC, SRL, and BEN were developed for this study and evaluated only in the sample used for the substantive analyses; the VDB items differ in content, the VC items could not be separated from acquiescence, and the practice items combined frequency stems with proportional response labels. Fifth, the survey used a convenience sample from a single Chinese university in which two-thirds of respondents were third-year undergraduates and 68.4% were men, and the first- and second-year group was small; the findings describe this institutional context and should not be generalized to computer-science students in China without further evidence (Yarkoni, 2022). Sixth, the interviews involved 11 self-selected, paid volunteers, 10 of whom were men, whose accounts could not be linked to their survey responses; the keyword-based unitization may have omitted relevant talk; and the coding was mainly carried out by one primary coder whose assignments were reviewed by the third author, so no inter-coder reliability coefficient is reported (O’Connor & Joffe, 2020). Future research should combine task-based verification measures, such as seeded defects in AI-generated code with confidence judgments, logged code traces and test construction, and objective assessments of code correctness and comprehension, with longitudinal or experimental designs that manipulate task stakes and the cost of checking, and should validate VDB with balanced keying in independent, multi-institutional samples.

## 7. Conclusions

This study examined which beliefs are associated with computer-science students’ reported practices for checking AI-generated code at one Chinese university. Using a concurrent mixed-methods design, survey data from 294 students were analyzed with an exploratory structural model and a set of robustness analyses, and interviews with 11 students were analyzed thematically and integrated with the survey results.

Three findings were most consistent. First, the breadth of GenAI delegation was unrelated to reported verification-related practices, so extensive AI use should not be treated as a proxy for weak checking. Second, verification-dispensability belief was the only construct negatively associated with all three practices across every robustness analysis, whereas generic self-regulation was associated with none and perceived AI literacy only with line-by-line comprehension. Third, the interviews showed that students allocated checking by task purpose, accepted process-level understanding, delegated checking to AI, and responded to institutional messages, practices that the survey captured only in part.

The study therefore suggests that beliefs about when checking is sufficient deserve attention alongside broader dispositions in research on AI-assisted programming. The central question is not only whether students use AI or possess general self-regulatory skills, but what evidence they treat as sufficient before accepting generated code.

At the same time, the study points to a limitation in current approaches to AI literacy and self-regulated learning. Both are often treated as broadly protective capacities, yet in this study neither was consistently associated with the reported checking of AI output. A learner can be highly self-regulated and confident with AI and still regard minimal evidence as sufficient. Future research should therefore move beyond self-report measures toward task-based verification studies that combine seeded code defects, objective diagnostic performance, confidence judgments, process traces, and experimental manipulation of task stakes and verification costs. Such work would help establish not only who verifies more, but when verification is warranted, what forms of evidence are sufficient, and how students can be taught to distinguish successful execution from justified acceptance.

## Figures and Tables

**Figure 1:**
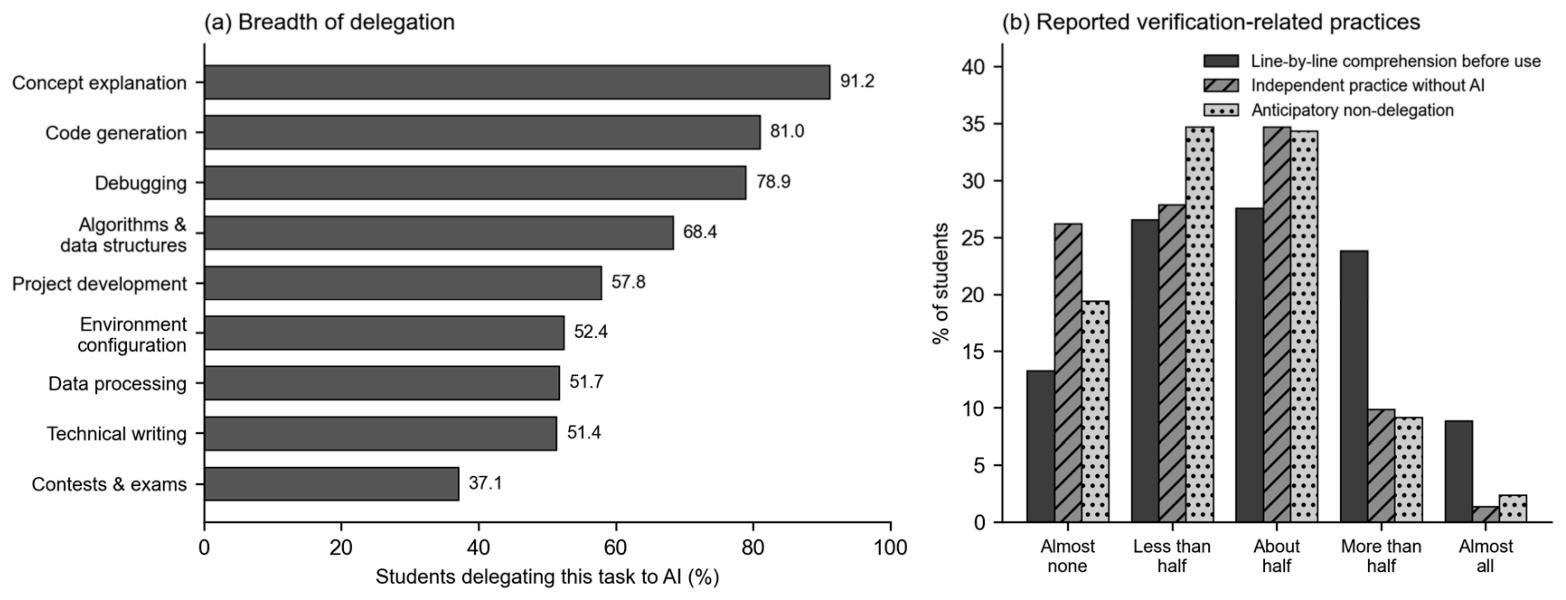
Breadth of GenAI delegation and distributions of reported verification-related practices.

**Figure 2:**
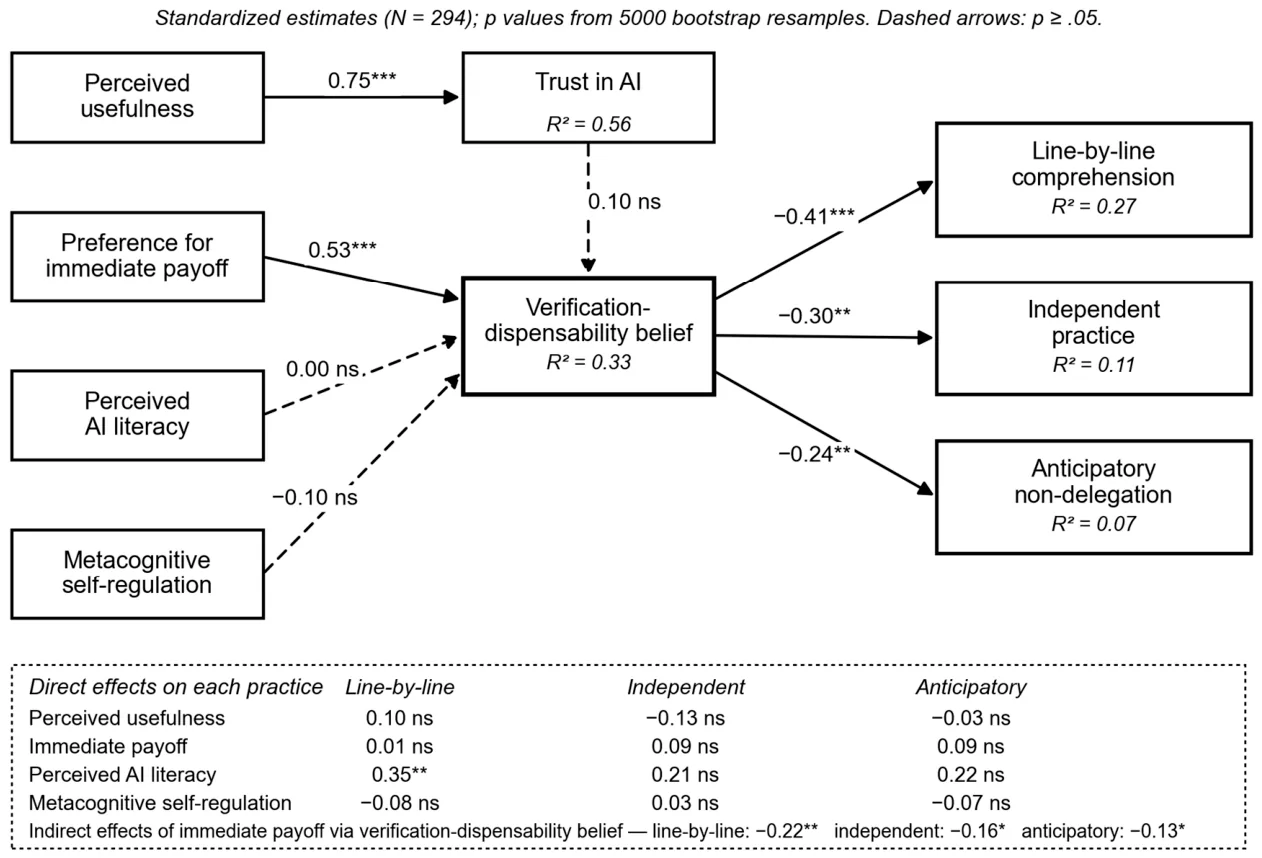
Exploratory structural model with the three practices as separate outcomes (standardized estimates). Solid arrows indicate paths with *p* < .05; dashed arrows indicate paths with *p* ≥ .05. * *p* < .05, ** *p* < .01, *** *p* < .001; ns = not significant (*p* ≥ .05).

**Table 1:** Measurement properties of the focal constructs.

Construct	Meaning	Items	Loading Range	ω	AVE
BEN	Perceived benefit (usefulness)	3	0.952–0.972	0.972	0.920
TRU	Trust in AI	2	0.948–0.949	0.947	0.899
IMP	Preference for immediate payoff	2	0.778–0.888	0.816	0.689
VDB	Verification-dispensability belief	3	0.714–0.844	0.842	0.640
VC	Verification commitment	3	0.635–0.815	0.780	0.544
AIL	Perceived AI literacy	3	0.904–0.943	0.949	0.860
SRL	Metacognitive self-regulation	3	0.856–0.910	0.915	0.780

Note. *n* = 294. Seven-factor CFA estimated with MLR; loadings are standardized. The three reported practices were single-item outcomes and are therefore not listed. AVE = average variance extracted.

**Table 2:** Gender and student-level differences in model variables and reported practices.

Variable	Men *M*	Women *M*	*d*	*p*	*q*	Year 1–2 *M*	Year 3+ UG *M*	PG *M*	*F*	η^2^	*q*
Verification-dispensability belief	4.34	4.01	−0.26	.037	0.334	3.93	4.33	4.05	1.99	0.014	0.358
Verification commitment	4.93	4.70	−0.23	.074	0.441	5.06	4.83	4.83	0.70	0.005	0.681
Perceived AI literacy	5.67	5.49	−0.18	.134	0.484	5.82	5.51	5.92	3.95	0.027	0.122
Metacognitive self-regulation	5.44	5.50	0.06	.645	0.794	5.64	5.37	5.73	2.89	0.020	0.178
Preference for immediate payoff	4.39	4.40	0.01	.957	0.971	4.14	4.47	4.27	1.31	0.009	0.611
Trust in AI	5.19	5.17	−0.02	.860	0.968	5.32	5.13	5.32	0.88	0.006	0.681
Perceived benefit	5.63	5.52	−0.10	.384	0.757	5.83	5.50	5.84	2.85	0.019	0.178
AI-related anxiety	4.10	4.57	0.34	.004	0.076	4.46	4.24	4.15	0.48	0.003	0.695
Line-by-line comprehension	2.91	2.80	−0.09	.469	0.757	3.48	2.71	3.17	8.35	0.055	0.005
Independent practice	2.31	2.38	0.07	.547	0.757	2.85	2.31	2.08	6.04	0.040	0.024
Anticipatory non-delegation	2.38	2.48	0.11	.385	0.757	2.48	2.37	2.54	0.73	0.005	0.681
Delegation breadth	5.53	6.05	0.19	.130	0.484	6.06	5.71	5.35	0.64	0.004	0.681

Note. Men *n* = 199, women *n* = 92; years 1–2 *n* = 33, year 3 and later undergraduates (UG) *n* = 210, postgraduates (PG) *n* = 48 (three respondents reporting another study level were excluded). *d* = standardized mean difference (women − men); *p* = Welch *t* test; *F* = one-way ANOVA with *df* = (2, 288); *q* = Benjamini–Hochberg-adjusted *p* across all 18 variables. Attitudinal variables were rated from 1 to 7, practices from 1 (almost none) to 5 (almost all), and delegation breadth from 0 to 9. Comparisons for all 18 variables are reported in Table S5.

**Table 3:** Standardized estimates from the exploratory structural model with separate practices.

Predictor	Trust in AI	VDB	Line-by-Line Comprehension	Independent Practice	Anticipatory Non-Delegation
Perceived benefit	0.749 (<0.001)	—	0.096 (0.171)	−0.127 (0.079)	−0.027 (0.719)
Trust in AI	—	0.105 (0.292)	—	—	—
Immediate-payoff preference	—	0.533 (<0.001)	0.011 (0.910)	0.091 (0.402)	0.089 (0.409)
Perceived AI literacy	—	0.002 (0.980)	0.353 (0.002)	0.207 (0.147)	0.221 (0.092)
Metacognitive self-regulation	—	−0.103 (0.298)	−0.076 (0.495)	0.027 (0.860)	−0.073 (0.598)
VDB	—	—	−0.408 (<0.001)	−0.301 (0.001)	−0.245 (0.007)
Indirect: IMP → VDB → practice	—	—	−0.218 (0.007)	−0.160 (0.015)	−0.130 (0.044)
Indirect: BEN → TRU → VDB → practice	—	—	−0.032 (0.341)	−0.024 (0.364)	−0.019 (0.385)
*R* ^2^	0.561	0.325	0.270	0.111	0.069

Note. *n* = 294. Entries are standardized coefficients with bootstrap *p* values in parentheses (5000 resamples); — = path not specified. Residuals of the three practices were allowed to correlate. Bias-corrected 95% confidence intervals for the unstandardized indirect associations via VDB were [−0.426, −0.096], [−0.288, −0.053], and [−0.261, −0.034] for line-by-line comprehension, independent practice, and anticipatory non-delegation. VDB = verification-dispensability belief; IMP = immediate-payoff preference; BEN = perceived benefit; TRU = trust in AI. Unstandardized estimates and confidence intervals for all paths are reported in Table S6.

**Table 4:** Robustness of the associations between beliefs and reported practices.

Association	Main Model *β*	WLSMV *β*	Single-Item VDB *β*	ACQ-Controlled *β*	Half-Samples (% *p* < .05)	Multiverse (% *p* < .05)
VDB → line-by-line comprehension	−0.408	−0.485	−0.395	−0.681	94.0	100.0
VDB → independent practice	−0.301	−0.328	−0.237	−0.831	73.4	100.0
VDB → anticipatory non-delegation	−0.245	−0.279	−0.180	−0.469	54.3	93.8
AIL → line-by-line comprehension	0.353	0.371	0.348	0.368	66.3	100.0
AIL → independent practice	0.207 ns	0.209 ns	0.202 ns	0.235 ns	17.1	40.6
AIL → anticipatory non-delegation	0.221 ns	0.247 ns	0.216 ns	0.245	24.1	37.5
SRL → line-by-line comprehension	−0.076 ns	−0.124 ns	−0.083 ns	−0.215 ns	3.0	25.0
SRL → independent practice	0.027 ns	0.066 ns	0.026 ns	−0.198 ns	5.5	31.2
SRL → anticipatory non-delegation	−0.073 ns	−0.052 ns	−0.073 ns	−0.181 ns	5.5	12.5
IMP → VDB	0.533	0.527	0.384	—	94.0	—
TRU → VDB	0.105 ns	0.229	0.100 ns	—	10.6	—
IMP → VDB → line-by-line comprehension	−0.218	−0.255	−0.152	—	70.9	—
IMP → VDB → independent practice	−0.160	−0.173	−0.091	—	50.3	—
IMP → VDB → anticipatory non-delegation	−0.130	−0.147	−0.069	—	25.1	—

Note. *n* = 294 except in the multiverse analysis, in which the screening rule varied (*n* = 244–366). Entries in columns 2–5 are standardized coefficients; ns = *p* ≥ .05. WLSMV = all indicators treated as ordered, with equal loadings for the two immediate-payoff items; single-item VDB = VDB represented by VDB1 (merging code without examination once tests pass); ACQ-controlled = latent regression including a random-intercept acquiescence factor and all model predictors (not estimated for paths to VDB or indirect associations). Half-samples = percentage of 200 half-samples (100 random splits) with *p* < .05; the sign matched the full-sample estimate in all half-samples for every VDB and indirect association. Multiverse = percentage of 32 specifications (4 screening rules × 4 covariate sets × 2 estimators) with *p* < .05. VDB = verification-dispensability belief; AIL = perceived AI literacy; SRL = metacognitive self-regulation; IMP = immediate-payoff preference; TRU = trust in AI; — = not applicable.

**Table 5:** Mixed-methods joint display.

Survey Result	Qualitative Theme (Units; Participants)	Relationship	Integrated Inference
Delegation breadth was unrelated to all three practices (*r* = −0.088 to 0.045)	Purpose-based allocation of checking (34; 11)	Qualification	Breadth conflates learning-oriented and delivery-oriented delegation; checking is allocated by task purpose, which a count of domains cannot show.
VDB was negatively associated with all three practices	Self-named illusion of competence (6; 5); running code treated as sufficient in low-stakes tasks	Confirmation	Students articulated the belief that further checking is unnecessary once code runs or the model appears competent.
Line-by-line comprehension was modest (*M* = 2.88 on a 1–5 scale)	Process-level redefinition of understanding (6; 4)	Qualification	Low scores partly reflect a different standard of sufficient understanding, not only an absence of checking.
The survey measured only comprehension achieved by the student	Delegated verification (30; 10) versus checking performed by the student (8; 5)	Conflict	Much checking was executed by AI, so reported line-by-line comprehension underrepresents checking and cannot indicate its quality.
Self-regulation was unrelated to all practices	Self-regulation serving efficient delegation (8; 4)	Confirmation	Regulatory skill can be directed toward efficient delegation as readily as toward checking.
Perceived AI literacy was associated with line-by-line comprehension only	Distinguishing declining production skill from retained judgment (8; 5)	Qualification	Perceived competence may relate to judging code rather than to maintaining unaided practice.
Institutional norms were not measured	Institutional legitimation (21; 9)	Extension	Thresholds for sufficient checking are partly set by course requirements and teachers’ messages.
Verification commitment could not be distinguished from acquiescence	Verification as a future professional role (16; 9)	Confirmation	Endorsing checking as a norm, in survey items or in interviews, did not indicate that checking was performed.

Note. Units = coded interview units; participants = number of the 11 interviewees who voiced the theme. VDB = verification-dispensability belief. The four relationship categories are defined in Section 4.5.

## Data Availability

The minimal dataset, the coding manual, the bilingual instrument, and the analysis scripts (R 4.5.2 and Mplus 8.3) supporting the reported results are available as Supplementary Files.

## Supplementary Materials

The following supporting information can be downloaded at: https://www.mdpi.com/article/10.3390/jintelligence14100232/s1, Table S1: Standardized factor loadings, seven-factor measurement model; Table S2: Reliability and convergent validity; Table S3: Discriminant validity: HTMT ratios and Fornell–Larcker criterion; Table S4: Latent correlations, means, and standard deviations; Table S5: Gender and student-level differences, all 18 variables; Table S6: Exploratory structural model with separate practices: unstandardized and standardized estimates; Table S7: Measurement comparisons: reflective practice factor and acquiescence; Table S8: Alternative estimations: all indicators ordered (WLSMV) and single-item VDB; Table S9: Associations with the practices before and after modeling acquiescence; Table S10: Split-half replication of the structural model (100 random splits); Table S11: Multiverse analysis: 32 specifications per association; Table S12: In-sample and cross-validated R^2^; Table S13: Exploratory specification search: models screened and multiple-comparison correction; Table S14: Unitization of respondent speech; Table S15: Theme frequencies and theme emergence across interviews; Table S16: Chinese originals and English translations of quotations; Section S1: Unitization rule for the interview corpus; Section S2: Coding manual (abbreviated); Section S3: Questionnaire items analyzed in this study (Chinese original and English translation).
