# Supplementary material for: Examining Computer-Science Students’ Metacognitive Beliefs and Reported Practices in Verifying AI-Generated Code: A Concurrent Mixed-Methods Study
Source: J Intell. 2026 Oct 1;14(10):232. doi: 10.3390/jintelligence14100232 (PMC13706013; doi:10.3390/jintelligence14100232)
Supplement: Supplementary file 1 [file jintelligence-14-00232-s001.zip › jintelligence-4562232-supplementary.pdf]

Supplementary Materials

Examining computer-science students' metacognitive beliefs and reported practices in verifying AI-generated code at a Chinese university: A concurrent mixed-methods study

Contents: Tables S1–S4 (measurement), Table S5 (group comparisons), Tables S6–S13 (structural model and robustness analyses), Tables S14–S16 (qualitative strand), Appendix A (unitization rule), Appendix B (coding manual), Appendix C (bilingual instrument). Abbreviations: VDB = verification-dispensability belief; VC = verification commitment; AIL = perceived AI literacy; SRL = metacognitive self-regulation; IMP = preference for immediate payoff; TRU = trust in AI; BEN = perceived benefit; ACQ = acquiescence factor.

Table S1. Standardized factor loadings, seven-factor measurement model

Construct	Item	Standardized loading	SE	p
Perceived benefit	BEN1	.952	.012	<.001
Perceived benefit	BEN2	.972	.012	<.001
Perceived benefit	BEN3	.954	.013	<.001
Trust in AI	TRU1	.948	.015	<.001
Trust in AI	TRU2	.949	.013	<.001
Preference for immediate payoff	IMP1	.888	.052	<.001
Preference for immediate payoff	IMP2	.778	.053	<.001
Verification-dispensability belief	VDB1	.714	.042	<.001
Verification-dispensability belief	VDB2	.844	.036	<.001
Verification-dispensability belief	VDB3	.842	.040	<.001
Verification commitment	VC1	.635	.059	<.001
Verification commitment	VC2	.777	.046	<.001
Verification commitment	VC3	.815	.054	<.001
Perceived AI literacy	AIL1	.943	.014	<.001
Perceived AI literacy	AIL2	.904	.026	<.001
Perceived AI literacy	AIL3	.935	.016	<.001
Metacognitive self-regulation	SRL1	.910	.024	<.001
Metacognitive self-regulation	SRL2	.856	.038	<.001
Metacognitive self-regulation	SRL3	.883	.030	<.001

Note. N = 294. Robust maximum likelihood (MLR). Item wording is given in Appendix C.

Table S2. Reliability and convergent validity

Construct	Cronbach's α	Coefficient ω	AVE	√AVE
Perceived benefit	.971	.972	.920	.959
Trust in AI	.947	.947	.899	.948
Preference for immediate payoff	.815	.816	.689	.830
Verification-dispensability belief	.838	.842	.640	.800
Verification commitment	.781	.780	.544	.738
Perceived AI literacy	.948	.949	.860	.927
Metacognitive self-regulation	.913	.915	.780	.883

Note. AVE = average variance extracted.

Table S3. Discriminant validity: HTMT ratios and Fornell–Larcker criterion

(a) Heterotrait–monotrait ratios

	BEN	TRU	IMP	VDB	VC	AIL	SRL
BEN	—						
TRU	.750	—					
IMP	.270	.476	—				
VDB	.147	.317	.600	—			
VC	.495	.394	.126	.446	—		
AIL	.599	.464	.065	.060	.413	—	
SRL	.430	.317	.094	.063	.388	.730	—

(b) Fornell–Larcker criterion

	**BEN**	**TRU**	**IMP**	**VDB**	**AIL**	**SRL**	**VC**
BEN	.959						
TRU	.747	.948					
IMP	.310	.495	.830				
VDB	.153	.317	.580	.800			
AIL	.597	.468	.106	.043	.927		
SRL	.424	.314	.004	−.061	.720	.883	
VC	.506	.392	.166	.455	.409	.373	.737

Note. In (b), diagonal entries are the square root of AVE and off-diagonal entries are latent correlations; no correlation exceeded the corresponding square root of AVE. All HTMT ratios were below .85.

Table S4. Latent correlations, means, and standard deviations

Variable	M	SD	1	2	3	4	5	6	7	8	9	10
1. Perceived benefit	5.59	1.07	—									
2. Trust in AI	5.18	1.10	.746	—								
3. Preference for immediate payoff	4.39	1.24	.306	.494	—							
4. Verification-dispensability belief	4.24	1.29	.149	.314	.585	—						
5. Verification commitment	4.87	1.04	.504	.392	.163	.449	—					
6. Perceived AI literacy	5.62	1.01	.597	.468	.100	.037	.412	—				
7. Metacognitive self-regulation	5.46	1.03	.424	.314	−.004	−.066	.378	.721	—			
8. Line-by-line comprehension	2.88	1.17	.213	.085	−.162	−.374	.143	.342	.246	—		
9. Independent practice	2.32	1.01	−.010	−.085	−.104	−.265	.182	.150	.143	.479	—	
10. Anticipatory non-delegation	2.40	0.98	.066	−.061	−.041	−.188	.096	.153	.092	.361	.543	—

Note. Correlations are from a CFA in which each practice was a single-indicator factor. Means and SDs are of observed scale scores (attitudes 1–7; practices 1–5).

Table S5. Gender and student-level differences, all 18 variables

Variable	Men M	Women M	d	t	p	q	Years 1–2 M	Year 3+ UG M	PG M	F(2, 288)	p	η²	q
Self-efficacy	5.29	5.20	−.08	−0.69	.490	.757	5.30	5.24	5.34	0.17	.839	.001	.839
Metacognitive self-regulation	5.44	5.50	.06	0.46	.645	.794	5.64	5.37	5.73	2.89	.057	.020	.178
Perceived AI literacy	5.67	5.49	−.18	−1.50	.134	.484	5.82	5.51	5.92	3.95	.020	.027	.122
AI-related anxiety	4.10	4.57	.34	2.89	.004	.076	4.46	4.24	4.15	0.48	.618	.003	.695
Fear of missing out on AI	4.47	4.64	.12	1.02	.308	.757	4.62	4.55	4.34	0.50	.607	.003	.695
Preference for immediate payoff	4.39	4.40	.01	0.05	.957	.971	4.14	4.47	4.27	1.31	.272	.009	.611
Perceived benefit	5.63	5.52	−.10	−0.87	.384	.757	5.83	5.50	5.84	2.85	.059	.019	.178
Trust in AI	5.19	5.17	−.02	−0.18	.860	.968	5.32	5.13	5.32	0.88	.414	.006	.681
Critical evaluation of AI output	5.62	5.57	−.05	−0.44	.662	.794	5.88	5.51	5.85	3.27	.039	.022	.177
Peer norms	5.58	5.45	−.13	−1.07	.284	.757	5.59	5.50	5.63	0.37	.689	.003	.729
Perceived accessibility and anthropomorphism	4.82	4.83	.01	0.04	.971	.971	4.57	4.85	4.90	0.92	.401	.006	.681
AI dependency	4.31	4.21	−.08	−0.65	.519	.757	4.21	4.33	4.10	0.68	.506	.005	.681

Verification-dispensability belief	4.34	4.01	−.26	−2.10	.037	.334	3.93	4.33	4.05	1.99	.139	.014	.358
Verification commitment	4.93	4.70	−.23	−1.80	.074	.441	5.06	4.83	4.83	0.70	.497	.005	.681
Line-by-line comprehension	2.91	2.80	−.09	−0.72	.469	.757	3.48	2.71	3.17	8.35	<.001	.055	.005
Independent practice	2.31	2.38	.07	0.60	.547	.757	2.85	2.31	2.08	6.04	.003	.040	.024
Anticipatory non-delegation	2.38	2.48	.11	0.87	.385	.757	2.48	2.37	2.54	0.73	.481	.005	.681
Delegation breadth	5.53	6.05	.19	1.52	.130	.484	6.06	5.71	5.35	0.64	.530	.004	.681

Note. Men n = 199, women n = 92; years 1–2 n = 33, year 3 and later undergraduates (UG) n = 210, postgraduates (PG) n = 48 (three respondents reporting another study level excluded). d = (women − men)/pooled SD; Welch t tests; q = Benjamini–Hochberg-adjusted p across the 18 variables in each family. Two one-sided tests with bounds of d = ±0.35 indicated equivalence across gender for line-by-line comprehension (p = .020), independent practice (p = .013), and anticipatory non-delegation (p = .024). Preference for immediate payoff is the validated two-item score.

Table S6. Exploratory structural model with separate practices: unstandardized and standardized estimates

Path	b	SE	p	95% BC CI (b)	β
Perceived benefit → Trust in AI	.743	.051	<.001	[.633, .832]	.749
Trust in AI → Verification-dispensability belief	.109	.103	.292	[−.095, .307]	.105
Preference for immediate payoff → Verification-dispensability belief	.518	.132	<.001	[.266, .786]	.533
Perceived AI literacy → Verification-dispensability belief	.003	.106	.980	[−.207, .206]	.002
Metacognitive self-regulation → Verification-dispensability belief	−.112	.108	.298	[−.322, .114]	−.103
Verification-dispensability belief → Line-by-line comprehension	−.432	.094	<.001	[−.620, −.254]	−.408
Perceived benefit → Line-by-line comprehension	.104	.076	.171	[−.042, .256]	.096
Preference for immediate payoff → Line-by-line comprehension	.011	.101	.910	[−.172, .223]	.011
Perceived AI literacy → Line-by-line comprehension	.416	.132	.002	[.140, .656]	.353
Metacognitive self-regulation → Line-by-line comprehension	−.088	.129	.495	[−.304, .190]	−.076
Verification-dispensability belief → Independent practice	−.275	.079	.001	[−.434, −.117]	−.301
Perceived benefit → Independent practice	−.119	.068	.079	[−.256, .008]	−.127
Preference for immediate payoff → Independent practice	.081	.097	.402	[−.078, .300]	.091
Perceived AI literacy → Independent practice	.211	.146	.147	[−.090, .483]	.207
Metacognitive self-regulation → Independent practice	.027	.154	.860	[−.229, .356]	.027
Verification-dispensability belief → Anticipatory non-delegation	−.217	.080	.007	[−.380, −.069]	−.245
Perceived benefit → Anticipatory non-delegation	−.024	.067	.719	[−.156, .104]	−.027
Preference for immediate payoff → Anticipatory non-delegation	.076	.092	.409	[−.078, .286]	.089
Perceived AI literacy → Anticipatory non-delegation	.217	.129	.092	[−.046, .449]	.221
Metacognitive self-regulation →	−.071	.134	.598	[−.295, .208]	−.073

Anticipatory non-delegation					
IMP → VDB → line-by-line comprehension	−.224	.084	.007	[−.426, −.096]	−.218
IMP → VDB → independent practice	−.143	.059	.015	[−.288, −.053]	−.160
IMP → VDB → anticipatory non-delegation	−.112	.056	.044	[−.261, −.034]	−.130
BEN → TRU → VDB → line-by-line comprehension	−.035	.037	.341	[−.113, .027]	−.032
BEN → TRU → VDB → independent practice	−.022	.025	.364	[−.078, .016]	−.024
BEN → TRU → VDB → anticipatory non-delegation	−.018	.020	.385	[−.068, .012]	−.019

Note. N = 294. Point estimates from maximum likelihood; standard errors, p values, and bias-corrected confidence intervals from 5000 bootstrap resamples. Residuals of the three practices were allowed to correlate.

(b) Model fit in lavaan and Mplus

**Software**	**χ^2**	**df**	**CFI**	**TLI**	**RMSEA [90% CI]**	**SRMR**
lavaan 0.6.21 (scaled)	223.49	126	.968	.956	.051 [.041, .061]	.052
lavaan 0.6.21 (robust)	223.49	126	.974	.965	.057 [.045, .069]	.052
Mplus 8.3 (MLR)	223.49	126	.968	.956	.051 [.040, .062]	.050

Note. lavaan robust indices follow Brosseau-Liard and Savalei; scaled indices use the conventional scaling correction reported by Mplus. The chi-square value and all standardized estimates were identical in the two programs.

Table S7. Measurement comparisons: reflective practice factor and acquiescence

**Model**	**Estimator**	**χ^2**	**df**	**CFI**	**TLI**	**RMSEA**	**SRMR**	**AIC**	**BIC**
Practices as one latent factor	MLR	268.11	136	.966	.957	.063	.064	13,125.2	13,324.1
Practices as separate outcomes	MLR	223.49	126	.974	.965	.057	.052	13,098.8	13,334.6
Practices as one latent factor	WLSMV	631.18	137	.986	.983	.111	.065		
Practices as separate outcomes	WLSMV	617.48	127	.986	.981	.115	.055		
VDB and VC, two factors	MLR	31.21	8	.945	.897	.128	.059	5428.2	5476.1
VDB and VC, two factors + acquiescence (VC2 residual fixed at 0)	MLR	14.72	8	.986	.974	.064	.031	5397.8	5445.7

**Comparison**	**$\Delta\chi^2$**	**Δdf**	**p**
Latent factor vs separate outcomes (MLR, Satorra-Bentler)	58.56	10	<.001
Latent factor vs separate outcomes (WLSMV, all indicators ordered)	54.81	10	<.001

Note. A reflective practice factor constrains every predictor's associations with the three practices to be proportional; the significant difference tests reject this constraint. In the WLSMV models all indicators were treated as ordered and the two immediate-payoff loadings were constrained to be equal, which prevented an improper negative residual variance for one of these items. The acquiescence factor had equal loadings on the six alternately keyed VDB and VC items and was orthogonal to both substantive factors. In the unconstrained acquiescence model the residual variance of VC2 was negative but not significantly different from zero (−0.32, 95% CI [−2.39, 1.76]); it was fixed at zero in all acquiescence models reported here and in Table S9.

(c) Acquiescence models: fit, VDB–VC correlation, and acquiescence variance

Model	χ^2	df	CFI	RMSEA	SRMR	BIC	r(VDB, VC)	Acquiescence variance
VDB and VC, two factors	31.21	8	.945	.128	.059	5476.1	.443	
Two factors + acquiescence (unconstrained; VC2 residual negative)	14.84	7	.984	.073	.031	5451.0	−.262	0.64 (p <.001)
Two factors + acquiescence (VC2 residual fixed at 0)	14.72	8	.986	.064	.031	5445.7	−.336	0.65 (p <.001)

Table S8. Alternative estimations: all indicators ordered (WLSMV) and single-item VDB

Path	WLSMV β	p	Single-item VDB β	p
Perceived benefit → Trust in AI	.792	<.001	.750	<.001
Trust in AI → Verification-dispensability belief	.229	<.001	.100	.275
Preference for immediate payoff → Verification-dispensability belief	.527	<.001	.384	<.001
Perceived AI literacy → Verification-dispensability belief	−.128	.125	.016	.852
Metacognitive self-regulation → Verification-dispensability belief	−.029	.708	−.137	.104
Verification-dispensability belief → Line-by-line comprehension	−.485	<.001	−.395	<.001
Perceived benefit → Line-by-line comprehension	.167	.087	.107	.105
Preference for immediate payoff → Line-by-line comprehension	.009	.891	−.058	.402
Perceived AI literacy → Line-by-line comprehension	.371	.004	.348	.001
Metacognitive self-regulation → Line-by-line comprehension	−.124	.234	−.083	.431
Verification-dispensability belief → Independent practice	−.328	<.001	−.237	<.001
Perceived benefit → Independent practice	−.152	.083	−.117	.093
Preference for immediate payoff → Independent practice	.132	.052	.008	.925
Perceived AI literacy → Independent practice	.209	.121	.202	.139
Metacognitive self-regulation → Independent practice	.066	.562	.026	.858

Verification-dispensability belief → Anticipatory non-delegation	−.279	<.001	−.180	.002
Perceived benefit → Anticipatory non-delegation	−.084	.367	−.018	.801
Preference for immediate payoff → Anticipatory non-delegation	.133	.058	.014	.865
Perceived AI literacy → Anticipatory non-delegation	.247	.084	.216	.089
Metacognitive self-regulation → Anticipatory non-delegation	−.052	.658	−.073	.596
IMP → VDB → line-by-line comprehension	−.255	<.001	−.152	.001
IMP → VDB → independent practice	−.173	<.001	−.091	.006
IMP → VDB → anticipatory non-delegation	−.147	<.001	−.069	.017
BEN → TRU → VDB → line-by-line comprehension	−.088	.001	−.030	.287
BEN → TRU → VDB → independent practice	−.059	.003	−.018	.300
BEN → TRU → VDB → anticipatory non-delegation	−.051	.005	−.013	.306

Note. In the single-item model, VDB was represented by VDB1 (merging code without examination once tests pass). WLSMV estimation treated all indicators as ordered, with equal loadings for the two immediate-payoff items; fit indices are reported in Table S7.

Table S9. Associations with the practices before and after modeling acquiescence

(a) Focused models with the six VDB and VC items

Model	Outcome	Predictor	β	SE	p
VDB only	Line-by-line comprehension	Verification-dispensability belief	−.354	.069	<.001
VDB only	Independent practice	Verification-dispensability belief	−.248	.074	.001
VDB only	Anticipatory non-delegation	Verification-dispensability belief	−.175	.073	.016
VDB + VC	Line-by-line comprehension	Verification-dispensability belief	−.541	.066	<.001
VDB + VC	Line-by-line comprehension	Verification commitment	.381	.076	<.001
VDB + VC	Independent practice	Verification-dispensability belief	−.430	.075	<.001
VDB + VC	Independent practice	Verification commitment	.374	.076	<.001
VDB + VC	Anticipatory non-delegation	Verification-dispensability belief	−.283	.078	<.001
VDB + VC	Anticipatory non-delegation	Verification commitment	.221	.076	.004
VDB + VC + ACQ	Line-by-line comprehension	Verification-dispensability belief	−.728	.195	<.001
VDB + VC + ACQ	Line-by-line comprehension	Verification commitment	−.201	.188	.286
VDB + VC + ACQ	Line-by-line comprehension	Acquiescence factor	.127	.091	.166
VDB + VC + ACQ	Independent practice	Verification-dispensability belief	−.529	.155	.001
VDB + VC + ACQ	Independent practice	Verification commitment	−.070	.153	.648
VDB + VC + ACQ	Independent practice	Acquiescence factor	.138	.090	.126

VDB + VC + ACQ	Anticipatory non-delegation	Verification-dispensability belief	−.382	.146	.009
VDB + VC + ACQ	Anticipatory non-delegation	Verification commitment	−.107	.144	.457
VDB + VC + ACQ	Anticipatory non-delegation	Acquiescence factor	.085	.092	.356
VDB + ACQ	Line-by-line comprehension	Verification-dispensability belief	−.562	.074	<.001
VDB + ACQ	Line-by-line comprehension	Acquiescence factor	.066	.082	.419
VDB + ACQ	Independent practice	Verification-dispensability belief	−.474	.069	<.001
VDB + ACQ	Independent practice	Acquiescence factor	.128	.077	.096
VDB + ACQ	Anticipatory non-delegation	Verification-dispensability belief	−.295	.071	<.001
VDB + ACQ	Anticipatory non-delegation	Acquiescence factor	.053	.077	.491

(b) Latent regression with all model predictors

**Model**	**Outcome**	**Predictor**	**β**	**SE**	**p**
covariates, no ACQ	Line-by-line comprehension	Verification-dispensability belief	−.372	.096	<.001
covariates, no ACQ	Line-by-line comprehension	Perceived AI literacy	.347	.110	.002
covariates, no ACQ	Line-by-line comprehension	Metacognitive self-regulation	−.065	.113	.565
covariates, no ACQ	Line-by-line comprehension	Trust in AI	−.011	.092	.902
covariates, no ACQ	Line-by-line comprehension	Preference for immediate payoff	−.017	.096	.860
covariates, no ACQ	Line-by-line comprehension	Perceived benefit	.105	.090	.244
covariates, no ACQ	Independent practice	Verification-dispensability belief	−.231	.094	.014
covariates, no ACQ	Independent practice	Perceived AI literacy	.200	.145	.169
covariates, no ACQ	Independent practice	Metacognitive self-regulation	.046	.159	.774
covariates, no ACQ	Independent practice	Trust in AI	−.128	.098	.192
covariates, no ACQ	Independent practice	Preference for immediate payoff	.066	.108	.540
covariates, no ACQ	Independent practice	Perceived benefit	−.035	.097	.716
covariates, no ACQ	Anticipatory non-delegation	Verification-dispensability belief	−.197	.089	.028
covariates, no ACQ	Anticipatory non-delegation	Perceived AI literacy	.228	.128	.074
covariates, no ACQ	Anticipatory non-delegation	Metacognitive self-regulation	−.062	.140	.659
covariates, no ACQ	Anticipatory non-delegation	Trust in AI	−.249	.110	.024
covariates, no ACQ	Anticipatory non-delegation	Preference for immediate payoff	.121	.100	.224
covariates, no ACQ	Anticipatory non-delegation	Perceived benefit	.136	.090	.134
covariates + ACQ	Line-by-line comprehension	Verification-dispensability belief	−.681	.200	.001
covariates + ACQ	Line-by-line comprehension	Perceived AI literacy	.368	.109	.001
covariates + ACQ	Line-by-line comprehension	Metacognitive self-regulation	−.215	.121	.074
covariates + ACQ	Line-by-line comprehension	Trust in AI	.065	.124	.597
covariates + ACQ	Line-by-line comprehension	Preference for immediate payoff	.158	.129	.221
covariates + ACQ	Line-by-line comprehension	Perceived benefit	−.252	.215	.241
covariates + ACQ	Line-by-line comprehension	Acquiescence factor	.043	.151	.777
covariates + ACQ	Independent practice	Verification-dispensability belief	−.831	.218	<.001

covariates + ACQ	Independent practice	Perceived AI literacy	.235	.147	.108
covariates + ACQ	Independent practice	Metacognitive self-regulation	−.198	.153	.196
covariates + ACQ	Independent practice	Trust in AI	.000	.171	.999
covariates + ACQ	Independent practice	Preference for immediate payoff	.350	.175	.045
covariates + ACQ	Independent practice	Perceived benefit	−.621	.257	.016
covariates + ACQ	Independent practice	Acquiescence factor	.324	.176	.065
covariates + ACQ	Anticipatory non-delegation	Verification-dispensability belief	−.469	.163	.004
covariates + ACQ	Anticipatory non-delegation	Perceived AI literacy	.245	.111	.027
covariates + ACQ	Anticipatory non-delegation	Metacognitive self-regulation	−.181	.125	.148
covariates + ACQ	Anticipatory non-delegation	Trust in AI	−.189	.126	.134
covariates + ACQ	Anticipatory non-delegation	Preference for immediate payoff	.264	.127	.038
covariates + ACQ	Anticipatory non-delegation	Perceived benefit	−.150	.178	.398
covariates + ACQ	Anticipatory non-delegation	Acquiescence factor	.103	.124	.406

Note. MLR estimation; models with the acquiescence factor fix the residual variance of VC2 at zero (Table S7). In (b), VC served only as indicators for identifying the acquiescence factor and did not predict the practices. A version in which VC also predicted the practices alongside all covariates and the acquiescence factor produced standardized coefficients exceeding 1 with inflated standard errors (empirical underidentification) and is not interpreted (log R1_03b).

Table S10. Split-half replication of the structural model (100 random splits)

Path	Full-sample β	Half-sample mean β	Half-sample SD	% p < .05	% same sign
BEN → TRU	.749	.747	.045	100.0	100.0
TRU → VDB	.105	.116	.102	10.6	89.4
IMP → VDB	.533	.522	.096	94.0	100.0
AIL → VDB	.002	.001	.094	1.0	53.3
SRL → VDB	−.103	−.104	.099	8.0	85.4
VDB → line-by-line comprehension	−.408	−.417	.099	94.0	100.0
VDB → independent practice	−.301	−.304	.088	73.4	100.0
VDB → anticipatory non-delegation	−.245	−.248	.086	54.3	100.0
AIL → line-by-line comprehension	.353	.340	.121	66.3	99.5
AIL → independent practice	.207	.182	.159	17.1	84.4
AIL → anticipatory non-delegation	.221	.193	.147	24.1	88.9
SRL → line-by-line comprehension	−.076	−.060	.110	3.0	70.9
SRL → independent practice	.027	.052	.159	5.5	55.8
SRL → anticipatory non-delegation	−.073	−.047	.162	5.5	65.3
IMP → VDB → line-by-line comprehension	−.218	−.219	.070	70.9	100.0
IMP → VDB → independent practice	−.160	−.158	.054	50.3	100.0
IMP → VDB → anticipatory non-delegation	−.130	−.130	.054	25.1	100.0

BEN → TRU → VDB → line-by-line comprehension	−.032	−.039	.038	7.0	89.4

Note. 199 of 200 half-samples (n = 147 each) converged.

Table S11. Multiverse analysis: 32 specifications per association

Outcome	Focal predictor	Median OLS β	Range of OLS β	% negative estimates	% p < .05
Line-by-line comprehension	VDB (three items)	−.419	−.541 to −.220	100.0	100.0
Line-by-line comprehension	VDB1 only	−.406	−.491 to −.266	100.0	100.0
Line-by-line comprehension	VDB without VDB2	−.395	−.512 to −.206	100.0	100.0
Line-by-line comprehension	Perceived AI literacy	.323	.211 to .380	0.0	100.0
Line-by-line comprehension	Metacognitive self-regulation	−.018	−.087 to .329	50.0	25.0
Line-by-line comprehension	Trust in AI	.052	−.065 to .160	18.8	12.5
Line-by-line comprehension	Preference for immediate payoff	.009	−.193 to .078	50.0	18.8
Line-by-line comprehension	Verification commitment	.201	.105 to .308	0.0	90.6
Independent practice	VDB (three items)	−.299	−.427 to −.134	100.0	100.0
Independent practice	VDB1 only	−.258	−.327 to −.150	100.0	100.0
Independent practice	VDB without VDB2	−.256	−.378 to −.121	100.0	100.0
Independent practice	Perceived AI literacy	.143	.043 to .213	0.0	40.6
Independent practice	Metacognitive self-regulation	.064	−.064 to .199	18.8	31.2
Independent practice	Trust in AI	−.088	−.115 to .000	100.0	3.1
Independent practice	Preference for immediate payoff	.097	−.143 to .203	25.0	37.5
Independent practice	Verification commitment	.358	.178 to .436	0.0	100.0
Anticipatory non-delegation	VDB (three items)	−.192	−.274 to −.084	100.0	93.8
Anticipatory non-delegation	VDB1 only	−.159	−.211 to −.049	100.0	65.6
Anticipatory non-delegation	VDB without VDB2	−.188	−.264 to −.101	100.0	93.8
Anticipatory non-delegation	Perceived AI literacy	.149	.084 to .223	0.0	37.5
Anticipatory non-delegation	Metacognitive self-regulation	−.013	−.181 to .169	43.8	12.5
Anticipatory non-delegation	Trust in AI	−.217	−.298 to −.013	100.0	71.9
Anticipatory non-delegation	Preference for immediate payoff	.109	−.073 to .162	21.9	15.6
Anticipatory non-delegation	Verification commitment	.161	.071 to .202	0.0	78.1

Note. Specifications crossed four screening rules (main rule, N = 294; all complete responses, N = 366; excluding respondents faster than half the median completion time, N = 265; excluding respondents who agreed strongly with both statements of a near-contradictory VDB–VC pair, N = 244), four covariate sets (none; model predictors; model predictors plus VC; all 18 measured variables), and two estimators (OLS with standardized variables; ordinal logistic regression). Percentages of negative estimates and of p < .05 are computed over all 32 specifications. VC estimates do not control acquiescence (see Table S9).

Table S12. In-sample and cross-validated R^2

Outcome	Predictor set	In-sample R^2	Cross-validated R^2 (mean)	SD
Line-by-line comprehension	VDB only	.132	.117	.005
Line-by-line comprehension	Model predictors without VDB	.152	.113	.005
Line-by-line comprehension	Model predictors	.262	.214	.012

Line-by-line comprehension	Model predictors + VC	.291	.242	.011
Line-by-line comprehension	All 18 measured variables	.316	.201	.013
Independent practice	VDB only	.064	.048	.005
Independent practice	Model predictors without VDB	.053	−.008	.015
Independent practice	Model predictors	.104	.038	.012
Independent practice	Model predictors + VC	.192	.130	.010
Independent practice	All 18 measured variables	.230	.089	.017
Anticipatory non-delegation	VDB only	.032	.017	.005
Anticipatory non-delegation	Model predictors without VDB	.051	−.008	.011
Anticipatory non-delegation	Model predictors	.080	.019	.011
Anticipatory non-delegation	Model predictors + VC	.100	.029	.013
Anticipatory non-delegation	All 18 measured variables	.150	−.014	.021

Note. Twenty repetitions of 10-fold cross-validation with ordinary least squares on observed scale scores. Model predictors = VDB, AIL, SRL, TRU, IMP, and BEN. Negative cross-validated values indicate prediction worse than the sample mean.

Table S13. Exploratory specification search: models screened and multiple-comparison correction

**Model family**	**Models**	**Raw p < .05**	**BH q < .05**	**Smallest q**
Single mediation	63	28	18	.000
Chain mediation	29	28	12	.000
Moderation	40	5	0	.067
Moderated mediation	84	15	0	.059
Total	216	76	30	.000

Note. Benjamini–Hochberg correction was applied across all 216 tests. All significant tests after correction were mediation tests; no moderation or moderated-mediation test survived correction. The retained model was additive.

Table S14. Unitization of respondent speech

**Interview**	**Retained**	**Excluded: < 25 characters**	**Excluded: no listed term**	**Excluded: formulaic or other**	**Respondent paragraphs**
01	38	11	7	0	56
02	33	4	1	1	39
03	30	6	2	1	39
04	33	6	2	1	42
05	30	14	0	1	45
06	34	7	2	1	44
07	44	3	3	1	51
08	35	12	3	1	51
09	39	9	3	0	51
10	35	3	3	1	42
11	38	10	3	0	51
Total	389	85	29	8	511
% of respondent characters	90.6	2.7	5.5	1.3	100.0

Note. Paragraphs of respondent speech as transcribed. The 29 excluded paragraphs with at least 25 characters but none of the listed terms were re-examined against the codebook; none described checking AI-generated code or the allocation of such checking, and all would have received the residual code.

Table S15. Theme frequencies and theme emergence across interviews

(a) Theme frequencies

**Code**	**Theme**	**Units**	**Participants (of 11)**
T1	Purpose-based allocation of verification	34	11
T3	Delegated verification (AI checks AI)	30	10

T5	Institutional legitimation	21	9
T7	Verification as future professional identity	16	9
T4	Self-regulation serving efficient delegation	8	4
T8	Distinguishing what degrades	8	5
T9	Verification actually performed by the student	8	5
T2	Process-level redefinition of understanding	6	4
T6	Self-named metacognitive illusion	6	5
T0	None of the themes	248	11
—	Unassigned	4	4

(b) New themes by interview, in the order in which interviews were conducted (29–31 July 2026)

Interview	Themes present	New themes	Cumulative themes
01	5	T1,T3,T6,T7,T8	5
02	3	T4,T5	7
03	8	T2,T9	9
04	7	—	9
05	3	—	9
06	6	—	9
07	7	—	9
08	5	—	9
09	5	—	9
10	7	—	9
11	6	—	9

Note. 389 coded units. All nine themes had appeared by the third interview.

Table S16. Chinese originals and English translations of quotations

Theme	Interview	Chinese original	English translation
Purpose-based allocation of verification	03	包括算法题或者上课时候的一些大作业，比如数据结构课程设计之类的，我会基本上全手搓，一点 AI 都没用过。第一时间想到 AI 的话，是比赛之类比较大型的项目，尤其是时间可能会很紧的时候，主要靠 AI。	For algorithm problems or large course assignments, such as the data-structures course project, I basically hand-code everything and have not used AI at all. AI comes to mind first for larger projects such as competitions, especially when time is tight; then I mainly rely on AI.
Purpose-based allocation of verification	07	算法竞赛相关代码，提交之前我一定会吃透全部逻辑，否则等同于作弊。前端这类我不擅长的内容，为了节省精力，我不会深究底层运行逻辑，只要求代码实现指定页面效果	For algorithm-competition code, I always fully understand all of the logic before submitting; otherwise it would amount to cheating. For things I am not good at, such as front-end work, I do not dig into the underlying logic, to save effort; I only require the code to produce the specified page effect.
Purpose-based allocation of verification	10	日常小型课程作业、简易项目会用 AI 辅助，里面大多是重复操作，AI 完成速度快，我只需要核对最终代码能否正常运行即可。	For everyday small course assignments and simple projects I use AI assistance; they mostly involve repetitive operations, AI finishes them quickly, and I only need to check whether the final code runs properly.
Process-level redefinition of understanding	06	AI 全权代编，就是只要求代码功能正常，完成后不查看内部逻辑，只做黑盒功能测试。AI 辅助编程，则是我需要完整理清代码流程、数据流向、业务逻辑	Having AI write everything means only requiring the code to work, not examining its internal logic afterwards, and doing only black-box functional testing. AI-assisted programming means that I need to work out the code's flow, data flow, and business logic in full
Process-level redefinition of understanding	11	不需要脱离 AI 独立完整复现代码，但要清晰理解代码每一部分的功能。	There is no need to reproduce the code completely on my own without AI, but I do need to understand clearly what each part of the code does.
Delegated verification (AI checks AI)	04	现在我们还有一套循环工作流，先用 GPT 生成代码，再用 Claude Code 审核代码对错	We now also have a loop workflow: first generate the code with GPT, then use Claude Code to check whether the code is right or wrong
Delegated verification (AI checks AI)	09	我写完程序后，会统一发给 AI 二次审核，工具会指出代码里的缺陷、潜在报错，我再根据提示逐一修改完善，审核通过后才会确认代码可用。	After I finish a program, I send all of it to AI for a second review; the tool points out defects and potential errors in the code, I fix them one by one following its prompts, and only after the review is passed do I confirm that the code is usable.
Self-regulation serving efficient delegation	02	如果现在 AI 写的代码跑不通，要找自己的原因，是不是哪里没弄对，不能怪 AI，现在 AI 写代码很牛。	If code written by AI does not run now, you have to look for the cause in yourself, whether you got something wrong; you cannot blame the AI. AI is very good at writing code now.
Institutional legitimation	02	老师说用 token 生成的就是保证质量的，我全程用 Claude Code 生成	The teacher said that whatever is generated with tokens is of guaranteed quality, so I generated everything with Claude Code
Institutional legitimation	07	老师表面明确禁止学生作业使用 AI，但实际几乎所有学生写 C 语言课程设计时都会借助 AI。	On the surface, the teacher explicitly prohibits AI in assignments, but in practice almost all students use AI when writing the C-language course project.
Self-named metacognitive illusion	01	很多人包括我，会有“AI 会了，等于我会了”这么一个错觉，忽略了对底层	Many people, including me, have the illusion that once AI knows it, I know it, and we neglect learning the underlying logic. AI has basically taken away our ability to judge whether code is correct.

		逻辑的学习。AI 让我们判断代码正确与否的能力基本上就是缺失了。	
Verification as future professional identity	11	看不懂代码、不做人工审查的 AI 生成代码绝对不能上线，这项筛选、审查能力会成为核心竞争力。	AI-generated code that cannot be understood and has not been reviewed by a person must never go live; this ability to screen and review will become a core competitive advantage.
Distinguishing what degrades	04	说实话，手写代码的实操能力确实退步了。但我的问题分析、顶层设计、解决复杂问题的思维能力一直在提升。	To be honest, my hands-on ability to write code by hand has indeed declined. But my problem analysis, top-level design, and thinking for solving complex problems have kept improving.
Verification actually performed by the student	03	我会把 AI 做的每一行变更都人工审查一遍，看到写得不合适的就回滚让它重写。	I manually review every line the AI changes, and when I see something written inappropriately, I roll it back and have it rewritten.
Verification actually performed by the student	05	代码最终运行结果正确，但内部逻辑存在错误，AI 只是强行算出最终数值。我通读代码内容的时候就能发现这类问题。	The final output of the code was correct, but its internal logic was wrong; the AI had simply forced out the final value. I can detect this kind of problem when I go through the code in full.

Note. Chinese originals are verbatim excerpts from the coded units. Translations were prepared by the third author with the help of generative AI and checked for meaning by the corresponding author. Ellipses in the manuscript mark omitted words within these excerpts.

Section S1. Unitization rule for the interview corpus

Respondent speech was divided into paragraphs as transcribed. A paragraph was retained as a coding unit when all three criteria were met:

- it was respondent speech (interviewer turns were discarded);
- it contained at least 25 Chinese characters;
- it contained at least one of the following terms:

(terms are given in the original Chinese) AI, ai, 代码, 核验, 检查, 审查, 校验, 读, 看懂, 理解, 逻辑, bug, 报错, 测试, 跑, 作业, 课程, 老师, 作弊, 底线, 依赖, 退化, 手写, 独立, 练, 验证, 信任, 准确

Paragraphs consisting of formulaic closings (self-introductions, thanks, farewells, bare assent) were additionally discarded. The rule yielded 389 coding units from 511 respondent paragraphs across the 11 participants (30 to 44 units per participant; mean length 97.6 characters). Excluded paragraphs are summarized in Table S14.

Section S2. Coding manual (abbreviated)

T1 Purpose-based allocation of verification

Definition. The speaker allocates checking according to the nature or purpose of the task (learning versus delivery; algorithm exercises versus projects; competition versus coursework).

Inclusion. A contrast between two task types, or an explicit statement of the form "in this situation I do, in that situation I do not".

Exclusion. Statements about how thoroughly code is checked without distinguishing task types (see T2).

T2 Process-level redefinition of understanding

Definition. The speaker redefines what counts as understanding: grasping the overall logic, execution flow, or data movement is treated as sufficient, without line-by-line examination or independent reproduction.

Inclusion. Formulations such as "I cannot follow the details but the flow is fine", or "knowing what each part does is enough".

Exclusion. Claims of complete or no comprehension without a stated standard (T0); handing comprehension to AI (T3).

T3 Delegated verification (AI checks AI)

Definition. Checking itself is handed to an AI system: asking the same model to inspect its own output, asking a second model to review it, cross-checking across models, or returning unclear code to a model for explanation.

Inclusion. A concrete practice in which AI is the executor of checking.

Exclusion. Sending an error message to AI for a fix without any indication that this replaces the speaker's own checking (T0); asking AI to explain a problem statement or concept rather than code (T0).

T4 Self-regulation serving efficient delegation

Definition. The speaker describes disciplined study habits (planning, attempting first, reflecting afterwards) that are directed at completing or delegating work efficiently rather than at scrutinising AI output, or that accompany a denial that AI errs.

Inclusion. A disciplined habit together with an efficiency orientation, or attribution of AI failure to the user rather than the system.

Exclusion. Attempting a problem before consulting AI where a task-type contrast is present (T1); practicing manual coding where capability decline is the topic (T8).

T5 Institutional legitimation

Definition. Requirements, encouragement, tacit permission, or explicit endorsement by teachers, courses, or the institution provide a warrant for selective non-verification.

Inclusion. A teacher, course, department, or institution must appear as the acting party.

Exclusion. Peer practice or peer norms (T0); the speaker's own integrity threshold with no institutional actor (T0).

T6 Self-named metacognitive illusion

Definition. The speaker names a mistaken sense of knowing, of the form "AI knows it, therefore I know it", or states directly that the capacity to judge whether code is correct is being lost.

Inclusion. An explicit self-identification of believing oneself to understand, or a direct statement that judgment is eroding.

Exclusion. Concern about capability decline without naming the illusion (T8).

T7 Verification as future professional identity

Definition. Reviewing, checking, or gatekeeping is described as the core or irreplaceable competence of computing professionals in the future.

Inclusion. A future-, employment-, or role-oriented statement whose substance is review or gatekeeping.

Exclusion. Accounts of the speaker's present checking (T1, T2); general claims about AI replacing jobs that do not reach review competence (T0).

T8 Distinguishing what degrades

Definition. The speaker separates two classes of ability, production or translation fluency on one side and comprehension, judgment, or design on the other, and states which is declining and which is not.

Inclusion. An explicit contrast between the two classes.

Exclusion. Undifferentiated statements that ability will decline (T0).

T9 Verification actually performed by the student

Definition. Checking that the speaker has personally carried out, with a human executor and an action that has actually taken place: examining each change line by line before committing, going through the whole file before submission, running tests and then inspecting affected modules, or rolling back and rewriting when a fault cannot be located.

Inclusion. A first-person habitual or past marker attached to an inspection act performed on code.

Exclusion. Checking executed by AI (T3); a standard of understanding with no accompanying action (T2); statements that verification ought to be done, or advice to others (T7 or T0).

T0 None of the above

Definition. The unit bears on none of the nine themes (tool inventories, program descriptions, privacy concerns, labour-market speculation, interview pleasantries).

Priority rule. Where a unit satisfies more than one definition, the code with the narrower inclusion criteria takes precedence: T6 > T5 > T3 > T9 > T8 > T7 > T4 > T2 > T1 > T0.

Rule clarifications applied during coding. (R1) Code or an error message handed to AI counts as T3 only where the text indicates that this substituted for the speaker's own checking; requesting a fix alone does not. (R2) The institutional actor in T5 is restricted to teachers, courses, departments, and the institution; employers and clients do not qualify. (R3) An allocation bounded by a deadline counts as T1 provided two situations are contrasted. (R4) T4 requires either a disciplined habit with an efficiency orientation or an attribution of AI failure to the user; both are not required. (R5) Definitional talk about assistance versus replacement that does not resolve into task type, standard of understanding, or an actual checking act is coded T0.

Section S3. Questionnaire items analyzed in this study (Chinese original and English translation)

Code	Construct	Chinese item	English translation	Use in the analyses
SE1	Self-efficacy	如果足够努力，我总能解决遇到的难题	If I try hard enough, I can always solve the difficult problems I encounter.	Group comparisons and robustness covariate
SE2	Self-efficacy	无论遇到什么，我通常都能想办法应对	Whatever happens, I can usually find a way to cope.	Group comparisons and robustness covariate
SE3	Self-efficacy	即使没有 AI 的帮助，我也有信心独立完成创作/学习任务	Even without help from AI, I am confident that I can complete creative or learning tasks independently.	Group comparisons and robustness covariate

SRL1	Metacognitive self-regulation	学习/创作时，我会先为自己设定明确的目标和计划	When learning or creating, I first set clear goals and plans for myself.	Main model
SRL2	Metacognitive self-regulation	遇到不会的地方，我会先尝试自己摸索，再寻求帮助	When I meet something I do not know, I first try to work it out myself before seeking help.	Main model
SRL3	Metacognitive self-regulation	完成作品/作业/任务后，我会反思哪里做得好、哪里需要改进	After completing a piece of work, an assignment, or a task, I reflect on what went well and what needs improvement.	Main model
AIL1	Perceived AI literacy	我知道如何向 AI 提出清晰、有效的指令（提示词）	I know how to give AI clear and effective instructions (prompts).	Main model
AIL2	Perceived AI literacy	我能判断 AI 生成的内容是否可用、是否需要修改	I can judge whether AI-generated content is usable and whether it needs to be modified.	Main model
AIL3	Perceived AI literacy	我能根据不同创作需求选择合适的 AI 工具或功能	I can choose suitable AI tools or functions for different creative needs.	Main model
ANX1	AI-related anxiety	AI 技术发展太快，让我感到不安	AI technology is developing so fast that it makes me feel uneasy.	Group comparisons and robustness covariate
ANX2	AI-related anxiety	我担心过度使用 AI 会削弱我自己的创作能力	I worry that overusing AI will weaken my own creative ability.	Group comparisons and robustness covariate
ANX3	AI-related anxiety	看到别人比我更会用 AI，我会感到压力	Seeing others use AI better than I do makes me feel pressured.	Group comparisons and robustness covariate
FOMO1	Fear of missing out on AI	如果不及时使用最新的 AI 工具，我担心会落后于同行	If I do not use the latest AI tools promptly, I worry that I will fall behind my peers.	Group comparisons and robustness covariate
FOMO2	Fear of missing out on AI	看到同学都在用 AI 提高效率/产量，我会担心自己吃亏	When I see classmates using AI to raise their efficiency or output, I worry that I will lose out.	Group comparisons and robustness covariate
FOMO3	Fear of missing out on AI	我害怕错过 AI 能带来的创作捷径或机会	I am afraid of missing the creative shortcuts or opportunities that AI can bring.	Group comparisons and robustness covariate
IMP1	Preference for immediate payoff	比起慢慢打磨，我更喜欢能立刻出效果的方式	Compared with slow refinement, I prefer approaches that produce results immediately.	Main model (items 1-2; item 3 excluded)
IMP2	Preference for immediate payoff	如果一件事需要长时间努力才有回报，我容易失去耐心	If something requires long effort before it pays off, I easily lose patience.	Main model (items 1-2; item 3 excluded)
IMP3	Preference for immediate payoff	我愿意为了更扎实的功底，放弃眼前的便利	I am willing to give up immediate convenience for more solid fundamentals. (reverse-worded; excluded)	Main model (items 1-2; item 3 excluded)
BEN1	Perceived benefit (usefulness)	使用 AI 能提高我的创作/学习效率	Using AI improves the efficiency of my creative work or learning.	Main model
BEN2	Perceived benefit (usefulness)	AI 能帮我更好地完成编程或识别漏洞等任务	AI helps me better complete tasks such as programming or identifying bugs.	Main model
BEN3	Perceived benefit (usefulness)	总体而言，AI 对我的专业学习很有帮助	Overall, AI is very helpful for my professional learning.	Main model

TRU1	Trust in AI	我相信 AI 生成的内容大体是可用的	I believe that AI-generated content is generally usable.	Main model (items 1-2; item 3 excluded)
TRU2	Trust in AI	我愿意依据 AI 的建议来推进我的创作	I am willing to advance my work on the basis of AI's suggestions.	Main model (items 1-2; item 3 excluded)
TRU3	Trust in AI	对于 AI 的输出，我会习惯性地审视、再加工	I habitually scrutinise and rework AI output. (excluded; content belongs to critical evaluation)	Main model (items 1-2; item 3 excluded)
CRIT1	Critical evaluation of AI output	采用 AI 生成的内容前，我会先判断它是否符合我的期望和目的	Before adopting AI-generated content, I first judge whether it meets my expectations and purposes.	Group comparisons and robustness covariate
CRIT2	Critical evaluation of AI output	我会把 AI 的产出与自己的判断相对照，而不是照单全收	I compare AI output with my own judgment rather than accepting it wholesale.	Group comparisons and robustness covariate
CRIT3	Critical evaluation of AI output	我能意识到 AI 生成在原创性、细节上可能存在的问题	I am aware that AI-generated content may have problems of originality and detail.	Group comparisons and robustness covariate
NORM1	Peer norms	我身边的同学大多都在用 AI 辅助创作/学习	Most classmates around me use AI to assist their creative work or learning.	Group comparisons and robustness covariate
NORM2	Peer norms	使用 AI 在我的同学圈里被视为很正常的事	Using AI is regarded as quite normal among my classmates.	Group comparisons and robustness covariate
NORM3	Peer norms	如果不用 AI，我会觉得和同行有点"脱节"	If I did not use AI, I would feel somewhat out of step with my peers.	Group comparisons and robustness covariate
ANTH1	Perceived accessibility and anthropomorphism	AI 工具随时可用，几乎没有门槛	AI tools are available at any time, with almost no barrier to use.	Group comparisons and robustness covariate
ANTH2	Perceived accessibility and anthropomorphism	与 AI 交流/共创时，感觉就像在和一个真实的人协作	Communicating or co-creating with AI feels like collaborating with a real person.	Group comparisons and robustness covariate
ANTH3	Perceived accessibility and anthropomorphism	有时我会对 AI 产生一种亲近或陪伴的感觉	Sometimes I feel a sense of closeness or companionship towards AI.	Group comparisons and robustness covariate
DEP1	AI dependency	如果不能用 AI，我会感到焦虑或不安	If I cannot use AI, I feel anxious or uneasy.	Group comparisons and robustness covariate
DEP2	AI dependency	没有 AI，我会觉得心里没底	Without AI, I feel unsure of myself.	Group comparisons and robustness covariate
DEP3	AI dependency	即使是自己能完成的创作，我也习惯先让 AI 出一版	Even for creative work I could complete myself, I habitually let AI produce a first version.	Group comparisons and robustness covariate
DEP4	AI dependency	我越来越难以在不使用 AI 的情况下完成创作任务	It is becoming harder for me to complete creative tasks without using AI.	Group comparisons and robustness covariate
DEP5	AI dependency	我倾向于直接采用 AI 生成的结果，而不再自己深入推敲	I tend to adopt AI-generated results directly without thinking them through further.	Group comparisons and robustness covariate
DEP6	AI dependency	我常常懒得去打磨 AI 的产出，直接拿来就用	I often cannot be bothered to refine AI output and just use it as it is.	Group comparisons and robustness covariate
VDB1	Verification-dispensability belief	只要测试全部通过，AI 写的代码我就直接合入，不再逐行细看。	As long as all tests pass, I merge AI-written code directly without examining it line by line.	Main model

VC1	Verification commitment	即使截止时间就在眼前，我也不会提交自己解释不清楚的代码。	Even when the deadline is imminent, I will not submit code that I cannot explain.	Measurement model and acquiescence analysis
VDB2	Verification-dispensability belief	现在写代码本来就靠 AI 补全，专门练手写没有多大必要。	Since code is now written with AI completion anyway, there is little need to practice writing code by hand.	Main model
VC2	Verification commitment	即使 AI 能立刻给出答案，刷题时我也先自己写出一版再对照。	Even when AI could give me the answer immediately, I first write my own version when practicing problems and then compare.	Measurement model and acquiescence analysis
VDB3	Verification-dispensability belief	正因为了解大模型的原理，我反而放心——它大多数时候是对的，不必每次都去验。	Precisely because I understand how large models work, I feel reassured: they are right most of the time, so there is no need to verify every time.	Main model
VC3	Verification commitment	我会按任务类型区别对待 AI 的输出：容易出错的类型必先核验，比较可靠的类型才直接用。	I treat AI output differently by task type: error-prone types are always verified first, and only more reliable types are used directly.	Measurement model and acquiescence analysis
VERIF	Line-by-line comprehension before use	过去一个月，AI 生成的代码中，我实际逐行读懂之后才使用的大约占：	In the past month, of the AI-generated code I used, the proportion that I actually understood line by line before using it was about:	Outcome (analyzed separately)
INDEP	Independent practice without AI	过去一个月，我不借助 AI 独立完成编程或算法练习的次数：	In the past month, the number of times I completed programming or algorithm exercises without the help of AI:	Outcome (analyzed separately)
AVOID	Anticipatory non-delegation	过去一个月，我因为预判"这类任务 AI 容易出错"而放弃用 AI、改为自己做或查文档的次数：	In the past month, the number of times I gave up using AI and did the task myself or consulted documentation because I anticipated that AI is error-prone on this type of task:	Outcome (analyzed separately)

Note. Attitudinal items: 1 = strongly disagree (非常不同意) to 7 = strongly agree (非常同意). Practice items (VERIF, INDEP, AVOID): almost none (几乎没有), less than half (不到一半), about half (约一半), more than half (大半), almost all (几乎全部). VDB and VC items were presented alternately in one block (Q23-1 to Q23-6). English translations were prepared by the authors with the help of generative AI.
